# Parliament, the Pandemic, and Constitutional Principle in the United Kingdom: A Study of the Coronavirus Act 2020

**DOI:** 10.1111/1468-2230.12753

**Published:** 2022-07-11

**Authors:** Pablo Grez Hidalgo, Fiona de Londras, Daniella Lock

## Abstract

Constitutions come under pressure during emergencies and, as is increasingly clear, during pandemics. Taking the legislative and post‐legislative debates in Westminster and the Devolved Legislatures on the Coronavirus Act 2020 (CVA) as its focus, this paper explores the robustness of parliamentary accountability during the pandemic, and finds it lacking. It suggests that this is attributable not to the situation of emergency per se, but to (a) executive decisions that have limited Parliament's capacity to scrutinise; (b) MPs’ failure to maximise the opportunities for scrutiny that did exist; and (c) the limited nature of Legislative Consent Motions (LCMs) as a mode of holding the central government to account. While at first glance the CVA appears to confirm the view that in emergencies law empowers the executive and reduces its accountability, rendering legal constraints near‐futile, our analysis suggests that this ought to be understood as a product, to a significant extent, of constitutional actors’ mindset vis‐à‐vis accountability.

## INTRODUCTION

Constitutions come under pressure during emergencies. That is true in the United Kingdom (UK) as it is elsewhere. While there is plentiful work on constitutional strain relating to war, counter‐terrorism, national security, and financial crises,^1^ the last two years have drawn scholars’ attention to the same theme in the context of public health emergencies and crises.^2^ This paper considers the role of Parliament vis‐à‐vis the Coronavirus Act 2020 (CVA 2020) as a context to explore and establish further the contours of such stresses, and to consider what, if anything, the pandemic context might tell us about the contemporary functioning of parliamentary accountability in the UK.

Taking as our central focus the legislative and post‐legislative debates in Westminster on the CVA 2020, and in Stormont, Cardiff and Holyrood on the LCMs on that Act, we explore the robustness of parliamentary scrutiny as a key mechanism of accountability. Broadening our analysis beyond Westminster to consider the legislatures of the three devolved jurisdictions allows us to explore whether and, if so to what extent, limitations in legislative scrutiny in Westminster might be mitigated in these settings. The significance of a four nations approach is highlighted by the role of the CVA 2020 in empowering the governments in Scotland and Northern Ireland with a suite of powers equivalent to those available for England and Wales in the Public Health (Control of Desease) Act 1984.^3^ Conscious of the temporalities of emergency law‐making,^4^ the complexity and scale of the CVA 2020, its lack of textual or substantive coherence,^5^ and the fact that, as Lord Bethell conceded, it was ‘drafted on the hoof’,^6^ we consider not only the passage of the CVA 2020 but also its subsequent periodic review in the House of Commons by means of ‘six‐month reviews’ (SMRs).^7^ This allows us to consider whether such *ex post facto* review was effective as a mode of compensatory scrutiny to mitigate the effects of ‘urgency’ in the passage of the Act.

Our analysis suggests that, with respect to the CVA 2020, parliamentary accountability has been lacking in significant ways during the pandemic. We contend that this is attributable not to the situation of emergency per se, but to three key factors identified through the analysis: (a) executive decisions that have limited Parliament's capacity to scrutinise CVA 2020 powers; (b) MPs’ failure to maximise the opportunities afforded by the SMRs to compensate in a substantive sense for limited legislative debate when passing the CVA 2020; and (c) the limited nature of LCMs as a mode of devolved parliaments holding the central government to account.

In presenting these factors, our analysis contributes not only to the growing body of literature on how the pandemic has impacted on parliamentary business in and beyond the UK,^8^ but also on how key actors’ disposition towards the principles of constitutionalism may play an important role in minimising or exacerbating COVID‐19's effects on the ordinary functioning of the UK constitution^9^ and, relatedly, on the role of law in emergencies and/or crises.^10^ While at first glance the CVA 2020 appears to confirm the view that, in emergencies, law empowers the executive and reduces its accountability so as to render legal constraints near futile,^11^ our analysis shows how this can be understood not as a product of law per se but, to a significant extent, of key actors’ ‘mindset’ vis‐à‐vis accountability.^12^

Ours is not the first study to express concerns about the extent to which Parliament has been marginalised and disempowered, and has itself been ineffective or non‐robust, in the pandemic. In an early intervention, Keith Ewing put it bluntly when he asked ‘Who governs Britain?’, noting that ‘ministerial statements in Parliament have given way to daily briefings by ministers (flanked by government experts) in an empty room with journalists, not MPs, on the receiving end remotely to ask questions. Broadcast live to the nation, the spectacle reinforces the marginalisation of Parliament … Absent a crisis, this would look like something straight from the populists’ playbook’.^13^ Meanwhile, in a joint letter to *The Times* on the 21 April 2021, representatives of the Constitution Unit at UCL, the Hansard Society, the Bingham Centre for the Rule of Law, and the Public Law Project characterised the extent of Parliament's marginalisation of the House of Commons as shocking.^14^

This concern with the marginalisation of parliament is to be expected in a constitutional system premised on the sovereignty of parliament and the related constitutional principle of parliamentary accountability.^15^ Of course, we know that there are practical disjunctures between pure constitutional principle and the reality of political life.^16^ As Lijphart classically put it, in Westminster systems governments are strong and oppositions weak.^17^ Political scientists have long established that ‘oppositions … rely on a majority – with presumably inimical incentives – to protect and extend their prerogatives’^18^ in the service of the broader constitutional good. Few maintain the fiction of *effective* parliamentary supremacy over the executive, even if such supremacy is a fundamental constitutional principle. In other words, we generally recognise and accept that parliament's ability to *exercise* its constitutional supremacy is in large part dependent on the willingness of the executive (and particularly an executive with a significant majority) to *allow* it to do so, and on its respect for parliament's constitutional role. Loughlin writes that ‘above all, [constitutional democracy] requires a culture that … recognises the need for restraint in the exercise of power’.^19^ This is precisely the sort of culture we refer to when we discuss the idea of constitutionalist mindset.

The paper proceeds in five further parts. We first outline in brief the CVA 2020, its passage, and its relationship to the parliaments in Northern Ireland, Scotland, and Wales. In doing so we surface the scale and significance of the powers contained in the CVA 2020 and their complex implications for human rights and executive power. We also outline the role and significance of the Northern Irish, Scottish and Welsh parliaments as scrutinisers of the CVA 2020, and explain our decision to mirror the ‘four‐nations’ approach to the pandemic reflected in the Act in our analysis. We then proceed to undertake substantive analysis across three parts, one focusing on legislative debates on the CVA 2020 in Westminster,^20^ one focusing on LCM debates in the parliaments of the devolved jurisdictions,^21^ and one focusing on the SMR debates in the House of Commons.^22^ We relied on the electronic version of the written record of speeches found in Hansard, the Scottish Parliament's Official Report, the Senedd's Record, and the Northern Ireland Assembly's Hansard. These speeches represent just under 28 hours of debate in total (19 hours 40 minutes in Westminster, four hours 30 minutes in Northern Ireland, three hours in Scotland, and one hour 15 minutes in Wales). We acknowledge that the text of these debates cannot tell us everything about how the CVA 2020 was made, consented to, and reviewed. In reality, only some of the work of passing and scrutinising legislation takes place in public debate in the Chamber. Side‐conversations, discussions between the government and opposition parties, inter‐governmental discussions with the Northern Irish, Scottish and Welsh governments, and policy and drafting deliberations are all important parts of the work of forming, proposing, and passing legislation.^23^ However, the processes that take place outside of the Chamber are, largely, difficult to access. Certainly, in the context of the CVA 2020 we know that there were discussions between the shadow secretaries for health and the government and between the different governments in the UK, because these discussions are referred to – in passing – in the Chamber debates themselves.^24^ However, we know little about the substance of those discussions, and can find out even less. Therefore, we focus for our analysis on the public record. Finally, we end the paper with a discussion of the broader implications of our analysis, for both UK constitutionalism and the broader debate on the role of law in emergencies.

The analysis that we present here is of course only partial; it focuses on just one piece of legislation, and it limits itself to one aspect of parliamentary work: the making of primary legislation. However, even through that narrow lens, long‐standing concerns about government control of the parliamentary timetable,^25^ the scheduling of contentious and complex debates right before recess or just before potential expiry of measures under a sunset clause,^26^ and the absence in parliament of ‘a corporate ethos which promotes collective functions such as accountability’^27^ emerge. They combine with a growing sense that the current government has what might charitably be described as a cavalier attitude towards constitutional principle^28^ to paint a worrying picture.

## THE CORONAVIRUS ACT 2020 AND THE ‘FOUR‐NATIONS’ APPROACH

The CVA 2020 is a complex, technical, and far‐reaching piece of legislation with significant constitutionalist implications.^29^ While our focus is on how the Act was made, rather than its content, it is appropriate to note the breadth of its provisions, which include powers to weaken duties of local authorities to provide social care,^30^ prevent mass gatherings from taking place,^31^ close educational and childcare facilities,^32^ postpone elections,^33^ and screen and detain potentially infectious persons.^34^ The CVA 2020 also includes temporary modifications of mental health legislation across the UK to reduce safeguards relating to compulsory and emergency admission,^35^ broad indemnities for health service activity,^36^ extensions to investigatory powers,^37^ and the introduction of video links in criminal proceedings.^38^ All of these powers have been exercisable by the passing of regulations, many introduced using the ‘made affirmative procedure’ (MAP).^39^ This requires that regulations be laid before parliament ‘as soon as reasonably practicable after being made’.^40^ Such regulations may be in force for 40 days without approval by parliament, following which they expire unless parliament approves them by resolution.^41^ Thus, the CVA 2020 is an enabling act that empowers Ministers and other public bodies across the United Kingdom to make (often very‐restrictive) regulations in response to the pandemic by means of ‘scrutiny‐lite’ procedures. As the regulations envisaged under the Act were always likely to be subject to limited pre‐ and post‐legislative examination given their status as delegated legislation and the complexity of the situation to which they would be responding, effective scrutiny of the CVA 2020 – including robust analysis of the rights‐compatibility of the types of regulations it enabled and foresaw at the time of its passage – was of particular importance.

The rights‐related implications of the CVA 2020, regulations made under it, and the pandemic per se are complex. On the one hand, when exercised powers in the CVA 2020 would interfere (albeit perhaps proportionately) with individuals’ rights, and the Government did not derogate from the European Convention on Human Rights (ECHR) or any other applicable international legal instrument in respect of them.^42^ On the other hand, the CVA 2020 formed part of a broader government response to a situation in which health and life were manifestly at risk. Given the Government's positive obligations to take appropriate steps to safeguard life under Article 2 of the ECHR^43^ and wide‐ranging obligations to ensure that health goods and services are available, accessible, acceptable and of high quality under the International Covenant on Economic, Social and Cultural Rights (ICESCR),^44^ the CVA 2020 can be cast as part of a rights‐protecting and rights‐fulfilling set of responses. The Act at once, then, limited and gave effect to rights, the key question being whether, at any given time or in any given set of circumstances, the CVA 2020 and its implementation struck a ‘fair balance’ between rights‐related imperatives that were at times in tension with one another. Such questions are of course central to much human rights adjudication and, in respect of qualified rights, are generally settled by reference to proportionality.

Given its constitutional role, parliament is among those institutional actors expected to consider the initial and continuing proportionality of the CVA 2020 as circumstances evolve over the pandemic. For this reason, our analysis of parliamentary debates focuses mainly, although not exclusively, on two benchmarks of good quality parliamentary scrutiny: engagement with rights and the rights‐related implications of the proposals being considered, and reliance on evidence.^45^ We identified indicia of rights framing by looking at express, implied or proxy references to rights. Indicia of evidence‐based interventions comprised of references to parliamentary committee reports and other materials produced by experts, academics, stakeholders, NGOs and other third sector organisations. There are three reasons why these benchmarks indicate quality legislative scrutiny: they enable parliamentarians (1) to recognise the wide range of rights‐related impacts of the response, (2) to engage substantively with questions of disproportionate impact on protected characteristics and socio‐economic status, and (3) to influence government responses by holding the state accountable for rights (non‐) compliance and insisting that rights‐limitations are temporary, proportionate, and fully rolled back once no longer necessary.

That parliament meets its responsibility in this regard is particularly important during periods framed as emergencies when courts are prone to deference^46^ and the usual stages and processes of legislation‐making are circumscribed or, in some cases, skipped in the service of ‘urgency’.^47^ We would expect the parliaments at Westminster, Cardiff, Edinburgh and Stormont to discharge their institutional responsibility of limiting and holding the UK governments to account^48^ in the pandemic context by seeking to ensure, during its passage, that the CVA 2020's provisions both enable an effective emergency response and are attentive to core constitutional principles, including rights and, after its passage, that this remains true in its implementation. It this thus apposite to explore whether the CVA 2020 was subject to rights‐framed and evidence‐based legislative scrutiny, especially as rights in the UK constitution do not operate as legal constraints on Westminster's legislative supremacy.

Although the CVA 2020 is not the only legislative framework for the pandemic response in the UK,^49^ it is an appropriate focus for our analysis of whether, how, and how well Parliament has executed that role since March 2020. The CVA 2020 is the only archetypical piece of emergency legislation that has been introduced in Westminster to deal with the pandemic.^50^ Thus, to the extent that the pandemic might be said to have introduced emergency‐like conditions or particular strains on Parliament, these are likely to have affected its passage in especially acute ways. Second, the CVA 2020 provided a reasonably stable statutory framework for the management of the pandemic over a period of two years from March 2020. Unlike regulations,^51^ it does not change rapidly or frequently, although the powers under it may be commenced or expired to different levels during its lifetime.^52^ The CVA 2020 thus offers a context through which to consider Parliament's behaviour over time, moving from the acute emergency of March 2020 through the different stages of the pandemic and across factors that introduced stability or disruption to the governance of the pandemic, such as increasing vaccine rollout, spikes and falls in case numbers, disease mutations and variants, increasing data and social and scientific evidence, and the global epidemiological and public health picture. Focusing on Parliament's engagement with the CVA 2020 since March 2020 thus allows us to move beyond the recognised constraints and imperatives of emergency and into a period of crisis management, when arguments for reduced scrutiny are more difficult to sustain.

The CVA 2020 was promulgated in Westminster to enable a four‐nation approach to the broad framework for the handling of the pandemic.^53^ In substantive terms this was achieved in the Act by both ‘levelling up’ devolved governments’ powers to make regulations under the types of powers contained in the Public Health (Control of Disease) Act 1984,^54^ and by creating a framework for devolved and non‐devolved powers across a wide range of policy areas.^55^ In March 2020 all eyes were on Westminster and on the legislation that would be introduced there. In this context it might be said that Westminster acted as the *de facto* scrutiniser for devolved nations. This is not to say that the devolved parliaments had no role. Although the ‘Sewel convention’ was operating very much as a facilitator in this context^56^ as the governments of the three devolved administrations had been consulted in the drafting of the CVA 2020 and supported the giving of consent, LCMs were introduced in each of these parliaments. It is well recognised that LCMs have a broad constitutional role; as a core part of the working of the Sewel Convention they contribute to ensuring that the UK has ‘a system for the exercise of legislative power … that [is] coherent, stable and workable’.^57^ While the parliaments in Northern Ireland, Scotland and Wales may be ‘an inferior political authority’^58^ to the parliament in Westminster, which retains superiority,^59^ they are nevertheless part of the broad accountability fabric of the UK and have the capacity, albeit limited, to scrutinise decision‐making by the UK government through these motions. By debating the LCMs on the CVA 2020 the parliaments in Northern Ireland, Scotland and Wales were, to an extent, also debating the CVA 2020 itself.

## LEGISLATIVE SCRUTINY AT WESTMINSTER

As a complex piece of legislation, one would ordinarily expect the CVA 2020 to receive a significant amount of time for debate and scrutiny.^60^ This was not the case. Instead, the Government used its control of the parliamentary timetable significantly to constrain the time available to debate the Bill and was remarkably closed to any attempts meaningfully to amend it, although it must be said that MPs were muted in their opposition and largely conceded to the rhetoric of urgent necessity so familiar in emergency law‐making contexts. While it made some concessions to future parliamentary accountability by means of providing for the SMR, this was – as becomes clear below – cosmetic at best. The CVA 2020 seems, at least on this reading, to be a typical piece of emergency law: quickly drafted, barely debated, and largely agreed to across the benches.

### Insufficient time

Parliamentarians have long complained that the executive is ‘in general too dominant over parliamentary proceedings’^61^ and ‘possesses an untrammeled power to decide the topics for general and topical debates’.^62^ In large part this is because the government has significant control over the time allotted for legislative debates and when such debates are scheduled.^63^ The passage of the CVA 2020 illustrates clearly how that government control can translate into very limited time for legislative scrutiny.

The Bill was published on 19 March 2020 with debate beginning four days later, on 23 March. The debate was scheduled for three days, finishing on the day Parliament rose for its Easter recess. The timing of the debate right up against recess of course curtailed the possibility of insisting on any extension. There was no realistic prospect that the entirety of the Easter recess – due to last until 21 April 2020 – would be allowed to pass without what the Government claimed was a vital part of the pandemic response being put in place.^64^ Therefore, the scheduled time of thirteen hours over three days was very clearly all Parliament would be allowed to debate this enormously complex Bill.

To understand just how brief a debating time this was, it is useful to think about it in context. Meg Russell and Daniel Gover have found that the average debating time is just over 75 hours per bill:^65^ more than four times what was allocated to the CVA 2020. Of course, the CVA 2020 was ‘emergency’ law so less time might be expected. Even then, however, the debating time was short. By means of example, the Anti‐Terrorism, Crime and Security Act 2001 (ATCSA), passed in the wake of 9/11 and pursuant to a derogation to the ECHR,^66^ received over 58 hours of debate, and parliamentarians had almost two months with the bill prior to that.^67^ In other words, the CVA 2020 received less than a quarter of the debating time of a piece of emergency law passed in a context where that was considered to be a ‘time of war or other public emergency threatening the life of the nation’.^68^ Furthermore, the CVA 2020 stretched to around 350 pages of complex, multi‐thematic legislation; the ATCSA came in at a ‘mere’ 124 pages, further suggesting that one might have expected more, rather than less, time for legislative debate.

It is also worth recalling that the hybrid parliament had not yet been introduced when the CVA 2020 was passed.^69^ Although no MPs raised concerns about this in the Chamber, there is every possibility that the debate being carried out ‘in‐person’ undermined some parliamentarians’ ability to participate. The debates began just days before the first lockdown in the UK, which was announced on 23 March, the same day the Bill finished its third reading at the Commons.^70^ By the time the Bill was passed, the UK was about to surpass ten thousand cumulative cases, and 1,000 daily cases.^71^ Many had already begun to self‐isolate including the Lord Speaker, Lord Fowler.^72^ There was, at that time, no facility for people who were not present to participate, which further limited in qualitative terms the debate on the Bill.

This is not to suggest that there was *no* debate or that MPs and Peers did not engage with the Bill. Although a crude measure, even just counting the number of parliamentarians who participated gives some indication of the Houses’ engagement. In the Commons, 56 MPs spoke at the Second Reading, and 25 spoke at the initial Committee Stage (of whom eight were new contributors). In the House of Lords, 37 Peers spoke in the Second Reading, 29 at Committee Stage (of whom 10 were new contributors), and five in Third Reading (of whom only one was a new contributor). In total, then, 64 of 650 MPs and 48 of 788 Peers spoke in these debates, so there was certainly considerable interest. However, it is not clear that people had time to make the interventions they would have wanted or, indeed, that there was sufficient time for everyone who wanted to do so to contribute. We do know that during the Second Reading of the CVA 2020, a five‐minute, and then four and three‐minute, limit on backbenchers' speeches was imposed for the final 105 minutes of the debate.^73^ No such limits were imposed in other debates, including in the Lords’ debates.

### Limited engagement

The limitations of the legislative debate were not confined to the time allocated. Our analysis also reveals that even those parliamentarians who did engage in the debates made fairly limited use of two important sources of scrutiny: evidence and human rights.

On the former, there was little engagement with concrete evidence regarding COVID‐19 itself or appropriate modes of pandemic response during the debates. At that time no parliamentary committees had reported on the pandemic, and there was a general lack of data on the disease, the emerging pandemic, and its impact on society. Thus, we might think it understandable that there would be only slight engagement with evidence. However, there was already at least some evidence on the basis of which to scrutinise government decisions reflected in the Bill, not least on PPE and testing. On the former, the WHO had by then published guidelines on PPE,^74^ which Jeremy Hunt MP (Conservative) urged the government to take account of.^75^ Furthermore, the then Shadow Secretary of State for Health, Jonathan Ashworth MP (Labour) and Munira Wilson MP (Liberal Democrat) both drew on media and anecdotal accounts of the shortage of PPE (Ashworth)^76^ and challenges posed by seeking to transfer patients from hospital to care homes without testing (Wilson)^77^ to seek to convince government to make better and further provision to limit transmission, albeit to little avail. Notably, however, evidence‐based interventions of this kind were rare.

Human rights made slightly more of an appearance in the debates, but rights‐based interventions were not generally successful in eliciting changes or concessions from government even when, as with Baroness Ludford's (Liberal Democrat) intervention, they relayed concerns from the Constitution Committee and the Delegated Powers Committee on the CVA 2020's potential implications for the right to life, the right to liberty, the right to family life, and the right to freedom of assembly and association.^78^ Across the debates we observe a number of different approaches to drawing on human rights. Some speakers referred generically to ‘human rights’ or ‘rights’,^79^ or used linguistic proxies for rights (especially references to ‘vulnerable groups’ as a proxy for discussion of *inter alia* non‐discrimination, the right to life, and the rights to health and education),^80^ while others made express but non‐specific references to the Human Rights Act 1998 or the ECHR.^81^ In the main these interventions made no express rights‐based ‘ask’ and instead sought to surface general concerns of the individual‐state relationship and rights‐related legality. In contrast, some speakers made very specific references to rights, raising concerns about the CVA 2020's implications for the rights of migrants,^82^ workers,^83^ disabled,^84^ young,^85^ and elderly^86^ people, rights to faith and dignity in death,^87^ to private and family life,^88^ and to protest.^89^ In some cases, parliamentarians offered concrete proposals to ensure effective rights protection, such as Zarah Sultana MP's (Labour) plea that ‘the Government release detainees from detention centres, suspend NHS charging, end data sharing between the NHS and the Home Office, and make sure that NHS staff and migrants know about it all with an information campaign’,^90^ or Ruth Jones MP (Labour)’s proposals that the Government ‘take some tangible steps now … underpin jobs and incomes with a comprehensive income protection scheme; … introduce European‐level statutory sick pay for all workers from day one; … tackle universal credit by increasing it, suspending sanctions and scrapping the five‐week wait for the first payment; and … assist the many millions of self‐employed people who are worried out of their minds about where their next penny is coming from’.^91^ Perhaps more striking is the range of manifest rights concerns that were not surfaced in the debates.^92^ For example, no consideration was given to the implications of the CVA 2020's weakening of safeguards on surveillance and data retention for the right to privacy,^93^ or to protecting the right to health by providing extra mental health support to families. This was despite the prospect of mass bereavements becoming clear and some parliamentarians expressing concern that mental health might decline as a result of lockdowns and school closures.^94^

### The refusal to amend

While one might point to ‘missed’ human rights issues in the debates, it is also important to acknowledge that where rights concerns were raised government made either no or, occasionally, very modest steps to address them. Indeed, this is part of a broader trend in the debate, where the Government generally refused to countenance amendments to the Bill.

MPs and Peers repeatedly raised concerns^95^ that changes to social care introduced in the CVA 2020^96^ would violate the rights of the elderly and those living with disabilities. However, the Government refused to support amendments to the CVA 2020 and offered no specific reassurances in the House of Commons on this issue other than to clarify that authorities were required to ‘follow’ the ECHR in exercising these powers,^97^ while in the Lords, Lord Bethell stated the Government would ‘do everything’ it could to ‘meet all needs’.^98^ Notably, Munira Wilson MP (Liberal Democrat) requested clarification as to the threshold for triggering the powers, and no response was given by the Government.^99^ When MPs raised concerns that changes to education provision^100^ would result in a lack of access to education by children with special educational needs,^101^ the Government provided no assurances and refused to support amendments to address this.^102^ The same was true in respect of MPs’ concerns that migrants and people in immigration detention would not have their right to health protected,^103^ and concerns already mentioned about frontline workers’ access to PPE.^104^ Reports published by independent public bodies and parliamentary committees would later confirm that these concerns were justified, indicating that the Government ought to have considered MPs’ rights‐based concerns more seriously.^105^

In fact, the only rights‐related amendments made were proposed by the Government in response to concerns raised in the (short) pre‐legislative window. The first related to concerns that the CVA 2020's requirement of cremation in cases of COVID‐related death would violate the right to freedom of religious belief,^106^ a concern the Government responded to by amending the CVA 2020 to ensure that people could be buried on religious grounds.^107^ The second related to concerns that the Bill contained no protections for renters affected by COVID‐19 who may be made homeless through eviction.^108^ The Government amended the Bill to extend the notice period for evictions by one month, resulting in a three‐month notice period,^109^ although MPs complained that this did not amount to the ‘eviction ban’ they claimed the government had promised in the pre‐legislative scrutiny phase.^110^ Even when asked to consider a general amendment, proposed in the Lords, to clarify that the exercise of the powers within the CVA 2020 would comply with the Human Rights Act 1998 and the Equality Act 2010,^111^ the Government refused. Lord Bethell stated he had been ‘advised by legal counsel’ that such an amendment was ‘potentially both unnecessary and unhelpful’^112^ as it ‘might imply that the Human Rights Act and Equality Act do not apply in this way in other Bills or Acts that do not feature this sort of provision’.^113^ This is notwithstanding the fact that a provision with a similar effect is found in at least one other piece of primary legislation and, indeed, in primary legislation on the pandemic in Scotland.^114^

As already noted, this reflects a broad trend whereby amendments were simply unwelcome. While 120 amendments were tabled at Committee Stage not all were debated because of a shortage of time,^115^ and ultimately all 23 amendments that were made to the Bill^116^ reflected new clauses submitted by the Government. This was partly a product of the Government's closed approach, but also partly reflected the limited opportunities for disagreement. At the first Committee Stage, the Chair of House of Commons Committee Stage, Dame Eleanor Laing, noted that the ‘bar’ for calling a division was ‘a high one’ so that arguments in favour of going through the Division Lobbies would need to be ‘very persuasive’.^117^ No divisions were called at this stage, and government amendments were prioritised.^118^ Of the 14 amendments referred to in the second Committee Stage in the House of Lords, five were withdrawn^119^ and nine were not moved.^120^ Of course, it is not uncommon for opposition amendments not to succeed or to be withdrawn,^121^ especially as the Government may table amendments that mirror Opposition demands. Overall, the approach of the Government was to introduce its own amendments rather than agree to those proposed by others. Notably, some subsequent developments in government policy, such as the creation of the Self‐Employment Income Support Scheme opened in May 2020, reflect proposals made in these rejected amendments.^122^ By and large, though, non‐Government amendments had very limited influence.

Perhaps more influential, but maybe only partially so, were concerns raised in pre‐legislative discussions. We have already noted that an amendment was introduced to allow religiously compliant burials following concerns being raised by MPs about original provisions for cremation. In addition, in welcoming the provision instating the SMRs^123^ MPs from across the House indicated that something of this kind had been the subject of discussion at the pre‐legislative scrutiny phase.^124^ A similar partial response to pre‐legislative concerns was evident in respect of tenants’ protections,^125^ which, as already mentioned, MPs considered to fall short of the ban on evictions they had anticipated.^126^

### Normal in an emergency?

Of course, one might respond to these illustrations of limited time, limited engagement, and very limited amendment by noting that the CVA 2020 is emergency law, passed when the Government was under time pressure due to the fast spread of COVID‐19 and the impending ‘lockdown’ due to be imposed on 26 March 2020. However, three important points should be borne in mind in this respect, all of which shed doubt on the extent to which an emergency that required new, primary legislation prevailed. First, the arrival of COVID‐19 in the UK was no surprise. The first detected case in the UK was recorded on 29 January 2020 and, while the WHO first declared a global pandemic on 11 March 2020,^127^ comparative experience already showed how serious a public health threat the disease was. There was no reason why legislation could not have been prepared sooner and in anticipation of the virus’ certain arrival, and the question of urgency is certainly not sufficient to explain the decision to schedule the debate right up against recess. Second, the ‘lockdown’ was imposed using the Public Health (Control of Disease) Act 1984 meaning the impending lockdown did not per se produce legislative urgency.^128^ Finally, and critically, some MPs and legal commentators argued that there was no clear need to introduce the CVA 2020 at all. The UK already had an established legislative framework in the form of the Civil Contingencies Act 2004 and the Public Health (Control of Disease) Act 1984 that, combined, could have provided sufficient legislative scaffolding for immediate action,^129^ with additive legislation (or even the CVA 2020 itself) being passed in the weeks and months that followed if needed and pursuant to a robust legislative process. Perhaps paradoxically, in passing the CVA 2020 in this way the Government might be said to have marginalised Parliament, even while seeming to place it front and centre in the pandemic response, by giving it inadequate time for scrutiny, creating a perception of absolute necessity that did not clearly exist, and scheduling the debate at a time and in a fashion that could only ever curtail its capacity for scrutiny. To adopt Lord Scriven's (perhaps understated) observation at the Lords Committee stage, this was ‘not the way to make good legislation’.^130^

## LEGISLATIVE CONSENT MOTIONS

Let us turn, now, to the devolved parliaments and the question of whether they have been an alternative source – albeit indirect – of accountability for the UK Government in respect of the CVA 2020. As we do so, it is worth recalling that all three parliaments are unicameral, and all have developed modes of working and procedures for legislative scrutiny that, combined with the particular devolution arrangements in each jurisdiction, insulate them to some extent from executive domination.^131^ Political realities are also germane. From the polarisation of Northern Ireland politics,^132^ to the Scottish National Party's minority and then coalition government,^133^ and the long‐standing pattern of minority or coalition government by Labour in Wales,^134^ there are good reasons to expect that the parliaments in Northern Ireland, Scotland and Wales might have been more capable of robust scrutiny, even though their capacity to scrutinise the UK government by proxy through LCMs is constrained by the form of the motion and the asymmetries of the UK constitutional order. Naturally, the devolved parliaments have a significant degree of structural independence from the government proposing the Bill to which they are being asked to consent, sometimes exacerbated by broader tensions about devolution or dissatisfaction with the impression that Westminster is ‘interfering’ in devolved matters. This is intensified by the different political make‐up of the devolved legislatures when compared to Westminster and, particularly, by the limited influence of the Conservative Party in the three devolved parliaments. Scotland is the only one of the three devolved parliaments in which the Conservative party was the main opposition party at the time of the passage of the relevant LCM. In Wales it was Plaid Cymru, and while the notion of ‘opposition’ is complex in the Northern Ireland Assembly,^135^ the Conservative Party has no parliamentary presence there.

That said, there was no real tension about the CVA 2020 from the perspective of devolution. Both the Scottish and Welsh governments referred expressly to their involvement in pre‐legislative discussions when presenting the LCMs and seeking consent.^136^ Furthermore, the Bill furnished the three devolved governments with new emergency powers,^137^ and therefore it is not surprising that they ‘recommended’ legislative consent. In the devolved legislatures, debates had a collaborative tone: no opposition amendment motions were debated, and in all three cases the LCMs were passed unopposed. The context also played its part. Broadly speaking, debates across the three different legislatures were characterised by the sense that COVID‐19 posed an unprecedented threat accompanied by a (somewhat reluctant) acceptance of the need for wide‐ranging, emergency executive powers, despite their human rights implications.^138^

### Short but sufficient time

In all three legislatures the parliamentary schedule is determined by a business committee and not, as in Westminster, by the government.^139^ However, for the LCMs on the CVA 2020, developments at Westminster conditioned the timing of debates and the legislatures certainly did not have a huge amount of time. In the three devolved legislatures, debates were scheduled and took place on 24 March, the day after the House of Commons completed its consideration of the Bill. In practice this suggests a limited amount of time to scrutinise the LCM. Furthermore, the three LCMs themselves were lodged at short notice.^140^ While there was in some cases limited time with the LCM, the CVA 2020 itself was of course published on 19 March and thus available to members of the three devolved parliaments from that point on.

While the LCM debates were short, there was no indication that they were *too* short and such debates are not usually very long. These motions are rarely contentious, and the scope for action is realistically narrow. Across the three parliaments there was no indication that parliamentarians felt unduly rushed or that they had insufficient time, and no unusual limits were placed on the duration of their interventions. In the Northern Ireland Assembly the debate ran from 10.30 am to four pm, with a one‐hour lunch break (ie for four and a half hours). The duration was determined by how much MLAs had to say: the Northern Irish Business Committee had not set a fixed limit on the extent of the debate, and so timing was not at issue. The Scottish Parliament debated the LCM for three hours. While the order of business did not specify the amount of time that would be devoted to the LCM in the Chamber, in practice it received one hour in the Chamber, 70 minutes at the Health and Sport Committee, and 50 minutes at the Finance and Constitution Committee. Finally, in Wales, the debate lasted for one hour and 15 minutes.^141^ In all cases the debates were held in person, rather than through a hybrid or remote process.^142^ In all three parliaments, the LCM was considered as a stand‐alone matter and was not bundled together with other COVID‐19‐related motions. Furthermore, on the day of the debate, agendas in the Welsh and Scottish parliaments were packed with other COVID‐19‐related business.^143^ There were, thus, ample opportunities for members of these two parliaments to participate more broadly in debates on the pandemic on the day. In both cases, the debates on the LCM were scheduled after COVID‐19 related statements by Welsh and Scottish ministers. Only in Northern Ireland was an amendment motion to the original LCM lodged, although this amendment was neither debated nor voted on.^144^

### Increased but still limited engagement

Given the limited nature of an LCM, the debates featured a perhaps surprisingly extensive range of evidence‐based interventions. MSPs, MSs and MLAs all relied on evidence produced by external bodies to flesh out their interventions, including on rights. These included a statement by the Equality and Human Rights Commission,^145^ a European Centre for Disease Prevention and Control's briefing to support emergency measures such as social distancing,^146^ concerns raised by NGOs about implications for the rights of vulnerable or marginalised groups,^147^ as well as concerns raised by constituents on matters such as the elderly living in care homes, experiences of those working in the industry and construction, and barriers experienced by disabled people in accessing care packages and education.^148^

In each of the three Devolved Legislatures, important steps were taken to try to ensure that relevant committees were active participants in the process of debating the LCMs.^149^ The most interesting of these committee engagements was in the Scottish Parliament where two committees held open evidence sessions in advance of the chamber debate.^150^ While the Finance and Constitution Committee questioned the Cabinet Secretary for the Constitution, the Health and Sport Committee questioned the Cabinet Secretary for Health and Sport. Committee members directed quite detailed questions to the Scottish Ministers. The Cabinet Secretary for the Constitution had to answer questions on how the Bill strikes a balance between human rights and public health policy aims, how vulnerable people are protected, including the elderly and people with mental health issues, how tenants would be protected in Scotland from eviction, and what relief measures would be in place for the self‐employed.^151^ The Cabinet Secretary for Health, on the other hand, had to answer questions on a variety of public health matters, including matters that went beyond the LCM debate, such as access to PPE and test and trace, and others concerning the LCM, such as the protection of mental health patients, including in the context of compulsory treatment orders, and protection of people subject to guardianship.^152^ Thus, these sessions involved scrutiny not only of what ‘local’ actions the Scottish government intended to take as part of its pandemic response, but also of the CVA 2020 itself and its implications for rights.

Although these oral evidence sessions may have not produced comparable evidence to that produced by external bodies or by such committees engaged in post‐legislative scrutiny, the evidence they did produce proved valuable, and the evidence sessions provided an opportunity for committee members to test in detail the knowledge and preparation of Scottish Ministers to deal with the pandemic in its early stages. There is also a stark contrast between the degree of generality of debates at the chamber and those that took place at committee where members focused on specific issues and asked detailed questions. This evidence had some but limited impact on the debate in the chamber. Given the proximity between the oral evidence sessions and the debate at the chamber, neither of the two committees had time to publish a report to inform the debate (their usual practice). The Presiding Officer gave five minutes each to the chairs of the Finance and Constitution Committee, and the Delegated Powers and Law Reform committees, to speak on behalf of their committees.^153^ Both chairs briefly outlined the reasons why they supported the LCM. Interestingly, in both cases, the chairs relied on ministerial assurances that the Scottish government would show self‐restraint in the exercise of the powers, ensure the protection of human rights and civil liberties, and use the MAP only in urgent cases.

The involvement of these committees added a certain structure and thematic focus to the LCM debates, but engagement with rights remained somewhat limited. Reflecting the nature of LCMs, there is a sense in the debates of inevitability of the enactment of the emergency measures even where rights are invoked, and most^154^ rights‐based engagements consist of general references to the negative impact of the emergency legislation on civil liberties,^155^ rights‐based expressions of concern about the potential for abuse of emergency powers,^156^ justifications of the emergency measures on the basis of necessity,^157^ proxy engagement of rights through references to generic or specific ‘vulnerable’ groups,^158^ or generally expressed concerns about ensuring financial protection and provision for individuals.^159^ Explicit references to human rights law, either international or domestic, were exceptional.^160^ Although parliamentarians expressed concern about the implications of emergency measures for ‘civil liberties’, in the majority of the interventions these were weighed against the unprecedented nature of the pandemic,^161^ and none of the devolved governments’ Legislative Consent Memorandums engaged meaningfully with the rights implications of the CVA 2020.^162^

Notwithstanding this, as in Westminster, a number of specific human rights issues were raised. Parliamentarians once again expressed concern about the impact that changes to social care would have on the rights of the elderly, children and those living with disabilities,^163^ and the effects of reducing safeguards for the detention of persons with mental ill‐health.^164^ In response to these concerns, the Northern Irish Minister for Health took the view that these powers were subject to appropriate safeguards,^165^ while his Scottish counterpart argued that these powers would be exercised only where absolutely necessary.^166^

### Government responsiveness … within limits

Given the institutional constraints on devolved legislatures, none of them had competence to shape the emergency response contained in the CVA 2020. However, parliamentarians did manage to exert some influence in terms of introducing bespoke review mechanisms on the exercise of the emergency powers conferred by the CVA 2020 at devolved level. Following representations from MSPs, the Cabinet Secretary for the Constitution in Scotland made commitments to report regularly to Parliament,^167^ and to incorporate provisions in forthcoming emergency legislation providing SMR‐type reviews and a two‐month reporting provisions analogous to those contained in the CVA 2020.^168^ Thus, the LCM debate served as an opportunity to secure both a ‘political’ commitment of accountability from the Scottish Government to the Scottish Parliament in respect of the CVA 2020 *and* a legislative commitment of formalised accountability processes in the then‐anticipated Scottish legislation, both of which commitments were fulfilled.^169^

Another Westminster development that had resonance at the Scottish Parliament was the introduction of protections for tenants from eviction in England and Wales. In committee session in the Scottish Parliament, Patrick Harvie MSP (Green) asked the Cabinet Secretary for the Constitution whether the government would ‘replicate the provisions that will apply to England and Wales – in effect, a ban on evictions’.^170^ Following commitments to this effect, extensive protections were included in the later Scottish Coronavirus Acts.^171^ In the Welsh debate on the LCM, MSs requested the government introduce similar reporting requirements as regards the operation of non‐devolved provisions.^172^ The Minister for Health made a political commitment to ‘reporting to this Assembly on the use of powers on a regular basis’,^173^ although this commitment was somewhat unspecific when compared with that provided in Scotland. Both governments have fulfilled these commitments.^174^

## POST‐LEGISLATIVE SCRUTINY IN WESTMINSTER

As we have seen, there were significant deficiencies in legislative scrutiny of the CVA 2020 in Westminster which could not fully be addressed by means of the LCM motions in the devolved legislatures. The SMR process was meant to serve as an ‘additional safeguard’ to ensure parliamentary accountability.^175^ However, in this section, we show that such reviews were severely lacking as a mechanism by which to hold the UK Government to account for the operation of the CVA 2020. First, this process was limited by the fact that MPs were only able to vote in favour of or against the Act in its entirety, as we have written about elsewhere.^176^ Moreover, despite repeated calls from MPs that the reviews should not be a rubber‐stamping exercise^177^ and that they must ‘provide meaningful scrutiny, protect human rights and promote public health’,^178^ the SMRs were defined by limited engagement with human rights and a lack of in‐depth, evidence‐based scrutiny of the operation of the CVA 2020.

### Enough time for a rubber stamp …

As with the legislative debates that preceded them, the UK Government exercised its control over the parliamentary schedule to greatly limit the potential for SMRs to act as accountability forums. First, both the first (SMR1) and third (SMR3) debates were scheduled for only 90 minutes each. When the allocation of 90 minutes was criticised during SMR1, then Health Secretary, Matt Hancock MP claimed that ‘[u]nder the Standing Orders of the House, this debate is 90 minutes, and neither the Speaker nor we had the choice over that’.^179^ This was a reference to Standing Order 16, which provides that the Speaker shall put questions necessary to dispose of a proceeding under any Act ‘not later than one and a half hours after the commencement of such proceedings’. However, the Speaker intervened: ‘the Secretary of State said that the time could not have been extended. Yes, it could, and I would have agreed to it’.^180^ This made it very clear that the Speaker did not consider the House bound to spend only 90 minutes on these reviews, putting the Government on notice that it could extend the debate further in future SMRs although, in SMR3, a mere 90 minutes was scheduled once again.^181^ By the time of this third review, the Government had proposed to expire or already expired 20 of the non‐devolved CVA 2020 powers.^182^ The second SMR (SMR2) was scheduled for 210 minutes in the House of Commons, with backbenchers being subject to a four‐minute limit for the last 165 minutes of the debate.^183^ By the time of SMR2, the Government had proposed that 12 of the non‐devolved CVA 2020 powers be expired.^184^ While on the face of it, this allocation of time seems an improvement over the first SMR, the Government scheduled the SMR2 motion to be debated in conjunction with three other motions,^185^ including a substantive and highly controversial motion on the Health Protection (Coronavirus, Restrictions) (Steps) (England) Regulations 2021.^186^ Although bundling multiple motions together meant that the putative 90‐minute limit understood to have been imposed by Standing Order 16 did not apply, it also restricted in practice the time Parliament could spend scrutinising the CVA 2020.^187^

Importantly, SMR1 and SM2 were conducted by the hybrid Parliament,^188^ meaning that parliamentarians could participate fully in proceedings even if self‐isolating, shielding or limiting their movements and close contacts as a public health measure. However, this could not resolve the problem of limited time. Across all three SMRs MPs criticised the paucity of time allocated for these debates. Charles Walker MP (Conservative) described a 90 minute debate as an ‘utter, utter disgrace’,^189^ and by SMR3 MPs seemed exasperated, with Dawn Butler MP (Labour) stating that ‘90 minutes every six months to scrutinise the Act really is not enough time for Parliament’^190^ and Munira Wilson MP (Liberal Democrat) lamenting that ‘once again we have been granted merely 90 minutes to discuss the remaining legislation’.^191^ The overall result of the Government's scheduling across the SMRs was that in eighteen months, MPs had 180 minutes of concentrated time to consider the operation of the CVA 2020 and 210 minutes of time considering that Act alongside other motions.

### (Still) limited engagement

While the absence of engagement with evidence might have been understandable during legislative debates on the CVA 2020, a plethora of evidence on which to base interventions existed for MPs to draw on in the SMR debates. These include two‐monthly reports mandated under the CVA 2020, which, although occasionally unreliable,^192^ and generally lacking in critical reflection on the effectiveness of the CVA 2020, did include a substantial amount of information on how the temporary powers were being used. However, these reports were not mentioned at all by MPs in the SMR debates, even though the House of Commons Library referred them to such reports in their briefings.^193^ Similarly, there were only two references to the One Year Report in SMR2.^194^

SMR debates also made extremely limited use of the extensive work on COVID‐19 done by Parliamentary Committees. None of these Committees’ reports were relied on in the debate on SMR1, there were only four references to such work in SMR2,^195^ and a mere three references in SMR3.^196^ This is particularly concerning given just how much scrutiny work parliamentary committees have undertaken since the beginning of the pandemic: 23 reports had been published by SMR1, a further 51 by SMR2, and a further 11 by the SMR3.^197^ Moreover, there were no references to important reports that have been produced by statutory bodies outside of Westminster (for example by the National Audit Office,^198^ the Equality and Human Rights Commission,^199^ and the Office of National Statistics^200^) and only a handful of mentions to reports from NGOs.^201^ Even with the limited time allocated to them, then, MPs seemed not to bring the extensive evidence available to them to bear on these SMR debates, undermining their potential effectiveness as an opportunity for scrutiny.

This was true also in respect of human rights, even though by the time of the SMRs acute concerns about the CVA 2020's rights‐related implications had arisen^202^ and relevant powers were not always slated for expiry.^203^ Indeed, human rights were only rarely mentioned: 14 times in the SMR1,^204^ 10 times in the SMR2,^205^ and just four times in the SMR3.^206^ In each SMR some critical human rights issues were missed, while others received only passing consideration. In SMR1, for example, MPs failed to press Government on the increasingly‐clear uneven impact of lockdowns on women,^207^ people suffering from domestic abuse,^208^ rough sleepers and precariously housed persons,^209^ those with pre‐existing mental health problems,^210^ prisoners^211^ and detainees,^212^ and people from BAME populations.^213^ Things barely improved in SMR2,^214^ when MPs failed to press Government on the uneven impact of school closures and lockdown on women,^215^ on children experiencing domestic abuse,^216^ and on children with parents in prison.^217^ Nor did they mention the lack of effective monitoring of support provided to clinically extremely vulnerable (CEV) people during the pandemic,^218^ and the disproportionate job losses faced by disabled people,^219^ and BAME populations^220^ during the pandemic. By the time of SMR3, MPs were raising rights‐related concerns with things not contained in the CVA 2020 at all (like vaccine passports),^221^ but still failing to challenge the government on prominent human rights concerns like the impact of video links in criminal proceedings on human rights,^222^ and the uneven impact that school closures under sections 37 and 38 of the CVA 2020 had on children across the UK.^223^

The point here is not to suggest that human rights concerns were never raised in the SMR debates; they were, sometimes in depth. However, it remains the case that significant human rights concerns – including ones that the JCHR and other committees had produced detailed reports on – were often and consistently ignored in these SMR debates. One implication is that critical human rights issues raised by the CVA 2020 were not debated in either legislative debates or the SMRs.

### Illusory by design

The limited time allocated to SMR debates and indeed MPs’ sometimes superficial engagement with rights concerns in some ways merely aggravated a greater problem: by design, the SMRs would only ever allow for illusory review of the CVA 2020. There are two reasons for this. The first is that only the House of Commons can vote on whether the temporary powers then in force should be expired or not; the CVA 2020 mandates that the motion on renewal/expiry should be laid before the Commons and that MPs shall vote on it, but prescribes no role for the Lords. While the House of Lords is statutorily mandated to debate a motion to take note of the one‐year status report, which took place contemporaneously to the SMR2,^224^ Peers have no opportunity to apply their extensive expertise in post‐legislative scrutiny to the CVA 2020 by means of SMR debates and motions. This exclusion was criticised at the time of the CVA 2020's passage, with Lord Newby calling it ‘unprecedented and completely unacceptable’.^225^ Nevertheless it was maintained.

Second, the form of the motion itself is limiting in the extreme. The Act prescribes that it should read ‘That the temporary provisions of the Coronavirus Act 2020 should not yet expire’. Clearly, this leaves little space for action. Through this motion, MPs are given the opportunity either to expire all or none of whatever temporary powers under the CVA 2020 are in operation at the time of the motion, but not to mandate that certain of these powers be expired while others be continued; it is an ‘all or nothing’ affair.^226^ In the SMRs the Government repeatedly reinforced this ‘all or nothing’ framing by suggesting that a vote to expire these provisions would have serious deleterious effects on individuals, including the loss of ‘measures protecting commercial tenants and renters from eviction, we would not be able to run virtual court hearings, which are an integral part of maintaining the rule of law, and people would not be able to receive statutory sick pay for the full period for which they are required to self‐isolate’.^227^ In other words, it framed a vote to expire as a nuclear option both for individuals receiving support under the CVA 2020 and for the country's pandemic response per se. This is notwithstanding the fact that a vote against the motion requires the Government to expire the powers within 21 days,^228^ so that there would be ample time for cross‐party discussions to identify which powers might immediately be re‐enacted or for other appropriate mitigations to be put in place and which could, in any case, be planned for *if* government engaged in meaningful cross‐party discussions *prior* to the SMR debate.

Despite its inclusion as a safeguard, then, the SMR was never going to be able to function as a mode of meaningful accountability and review; it simply was not designed to. This does not mean that the existence of the SMR had no effect. Certainly, it is plausible that the pressure of the semi‐regular reviews created a stable timetable for government decisions to expire or not temporary provisions of the CVA 2020. Indeed, within eighteen months of its commencement half of the non‐devolved CVA 2020 powers had been expired, and their status was regularly updated in the two‐monthly reports required by the Act.^229^ However, there was no indication of the Government seeing these reviews as moments for reconsideration of the approach taken in the CVA 2020, even when – as in SMR2 – they were presented by the Opposition with a proposal for a new approach to governing the pandemic. The Opposition Bill was introduced days before the SMR2 debate by Dawn Butler MP (Labour), and was based on a draft Bill produced by human rights NGO, Liberty.^230^ During SMR2 a number of MPs expressed support for it, but the Government did not even acknowledge its existence in its response to MPs at the end of the debate.^231^

## ANALYSIS

In principle there is nothing novel about a piece of emergency legislation exposing the stresses and institutional limitations of Parliament. Emergencies disempower parliaments, both because of a popular demand for ‘something to be done’ and a political imperative to ‘support’ the government's judgment as to what that something is.^232^ We know that legislation introduced in response to a perceived emergency tends to be passed quickly, with limited scrutiny, and often with very far‐reaching implications. Parliamentary scrutiny is often strained in these situations, and *ex post facto* mechanisms like periodic review, sunset clauses and even independent reviews are sometimes inserted in legislation as concessions with no realistic prospect that they will lead to any change in government approach or hasten the end of implicated powers at anything other than the government's own timetable.

The introduction and review of the CVA 2020 appears to align with some scholars’ view that emergency law‐making inevitably results in a lack of executive accountability,^233^ producing what Dyzenhaus calls ‘legal grey holes’. He defines these as legal spaces where there are ‘some legal constraints’ on executive action, which renders them ‘not a lawless void’, though such constraints are ‘so insubstantial that they pretty well permit the government to do as it pleases’.^234^ In other words, there is the appearance of scrutiny, but little actual scrutiny in practice. This clearly resonates with the CVA 2020 and its review processes and is exacerbated by what has been a deferential judicial approach to the scrutiny of powers under the CVA 2020.^235^ A further trend familiar to emergency scholars – normalisation – is also already in evidence, with some CVA 2020 powers becoming permanent. ‘Temporary’ powers to use video links in criminal proceedings were made permanent by means of the Police, Crime and Sentencing Act 2022,^236^ and the Government also made permanent powers to suspend inquests with a jury where COVID‐19 is suspected as the cause of death in the Judicial Review and Courts Act 2022.^237^ This is not unique to the central government: the Scottish Parliament is currently discussing the Coronavirus (Recovery and Reform) (Scotland) Bill, which seeks to make a number of these powers permanent.^238^

Despite this, we contend that the mere invocation of ‘emergency’ is not sufficient to explain *why* these trends are apparent in respect of the CVA 2020. Emergencies do not produce poor process, light touch engagement with rights, or an aversion to scrutiny. Instead, they are contexts in which inclinations towards such behaviours can be indulged and may even be tolerated; they are hard tests of constitutional actors’ commitments and dispositions. Rather than understanding the CVA 2020 as just another emergency law, we read it as an exposition of how important a meaningful commitment to accountability on the part of the executive, MPs and the devolved legislatures is to maintaining constitutionalism in and beyond crisis contexts.

### Executive decisions

As we have seen, where it has made decisions about Parliament's involvement in the passage and scrutiny of the CVA 2020 the Government has acted to limit that involvement. In timetabling the legislative debate for the CVA 2020 and then the SMRs, the Government gave parliamentarians a derisory amount of time. Moreover, the initial legislative debate was scheduled so close to Easter recess that Parliament was essentially given no choice but to pass the bill or be left without specific COVID‐19 legislation as it went into recess (and cancelling or curtailing the recess was not seriously contemplated), during which the first wave of the pandemic was underway. It placed Parliament on a cliff edge: pass this legislation or leave the state without the tools purportedly needed to suppress the pandemic and support people during lockdown. Meanwhile the sufficiency of existing legislative frameworks seems hardly to have been considered: something had to be done, and that something was the CVA 2020. Even when seeming to concede that *ex post facto* scrutiny might be needed in the form of periodic reviews, Government designed the SMRs in such a way as to almost guarantee their impotence, paying no attention to MPs’ concerns that their inability to amend the motion and to expire only some of the powers in force would have precisely this effect.

The Government, in other words, never conceded power to Parliament: it used and maintained its numerical and procedural dominance to minimise the extent to which Parliament could challenge, scrutinise, and seek to exert its own authority in respect of the CVA 2020, manifesting apparent disregard for the principle of Parliamentary accountability.

### MPs’ failures

It is hardly fair, though, to ascribe the blame entirely to the executive. If Parliament is supreme partly because the executive recognises and concedes to this, then the executive is dominant partly because Parliament allows it to be. We have seen that MPs failed to maximise the opportunities afforded by the SMRs to compensate in a substantive sense for limited legislative debate when passing the CVA 2020, just as they largely struggled to bring substantial human rights concerns to bear on the legislative debates themselves, thus failing effectively to fufil Parliament's limiting function within the UK's constitutional order.^239^ Most striking, perhaps, is MPs’ neglect of the extensive evidence available to them in both the UK Government's two‐monthly reports on the operation of the CVA 2020 and, crucially, the reports of parliamentary committees and external statutory bodies. By ignoring committee work, MPs failed meaningfully to connect what happens in committee with what happens in the Chamber. This both reflected and compounded their lack of rigorous engagement with human rights issues, either by entirely neglecting human rights concerns (including those on which committees had reached clear conclusions),^240^ or by making vague and unspecific interventions on rights instead of using reports and NGO or independent agency findings to concretise claims and develop evidence‐based scrutiny of the necessity, effectiveness, and broader impacts of the powers applied under the CVA 2020.^241^

MPs’ failure to maximise the opportunites for scrutiny at the legislative and post‐legislative stages and lack of engagement with human rights issues had concrete consequences throughought the pandemic.^242^ This can be illustrated by reference to the right to protest. Section 52 and Schedule 22 of the CVA 2020 granted powers to prohibit or otherwise restrict events or gatherings in England, Scotland, Northern Ireland and Wales. During the passage of the CVA 2020 at Westminster, the then Shadow Secretary for Health, Jonathan Ashworth, raised concerns that these powers ‘could impede the ability to protest’.^243^ However, MPs did not pass amendments to tighten up these powers with a view to uphold the right to protest, leaving this potentially serious human rights issue unaddressed. The later cases of *R (Dolan)* v *Secretary of State for Health and Social Care* 244 (*Dolan*) and *Leigh and others* v *The Commissioner of the Police of the Metropolis and other* 245 (*Leigh*) shed light on the limited ability of courts to ‘address gaps’^246^ arising from such lack of scrutiny. *Dolan* made clear that judicial review was of very limited usefulness in the pandemic since the fast‐changing nature of lockdown regulations would render challenges to the compatibility of regulations with human rights largely ‘academic’.^247^ Unsurprisingly, *Dolan* reinforced courts’ deferential posture in cases where substantive review does take place.^248^

The limitations on the capacity of courts to ‘address the gaps’ became apparent in *Leigh* when a campaign group sought the intervention of the Court for the purposes of organising a vigil for the late Sarah Everard while London was subject to Tier Four restrictions prohibiting mass gatherings. They sought urgent interim relief in the form of a declaration that regulations under the CVA 2020 must be read and applied compatibly with the Human Rights Act 1998 and, consequently, that ‘persons who are exercising their right to protest in a reasonable manner will have a reasonable excuse for gathering’ and thus be permitted to protest. The Court took the view that clarifying the scope of the ‘reasonable excuse’ defence in the terms sought by the applicants would be ‘inaccurate’ and ‘potentially misleading’.^249^ The Metropolitan Police confirmed its view that attendance at any vigil would be unlawful under the regulations then in place and warned that organisers could face fines of up to £10,000 each and possible prosecution. The official vigil was cancelled.

A couple of days after this judgment was delivered, during the SMR2 debates, the shadow Secretary came back to the issue of the right to protest, expressing the view that Schedule 22 was ‘open to abuse’ and that he expected that the Government would ‘review it and come forward with alternatives’.^250^ However, the Government did not face major pressure from Parliament to introduce additional safeguards to uphold the right to protest. The High Court has recently confirmed that the Metropolitan Police's approach to the vigil violated the organisers’ rights to freedom of expression and freedom of assembly as the police force failed to perform its legal duty to consider whether the organisers might have had a reasonable excuse for holding the gathering or to undertake a concrete proportionality assessment required to perform that duty.^251^ However, as is usual in such cases, this decision came about a year after the events took place underlining the irreplaceable role of strong, evidence‐based and rights‐framed scrutiny of government legislation during the legislative process.

### The limits of the LCM

LCMs are a critical instrument in the relationship between central and devolved government and legislatures, but the CVA 2020 exposes the ways in which their in‐built limitations (which in turn reflect the compromises of the multi‐level constitution) constrain the capacity for devolved legislatures to hold central government to account. In part this reflects what LCMs enable devolved parliaments to do: to consent, or not, to legislation proposed in Westminster, not to change it. With respect to the CVA 2020, politics and context also played a role. The timing was not unimportant. As already mentioned, the devolved Governments scheduled the LCM on 24 March 2020, one day after the CVA 2020 had completed all of its Commons stages. This departed from standard practice: LCMs are usually decided on before a Bill reaches its final amending stage at the House of introduction^252^ so that the UK Government has an opportunity to address the concerns expressed by devolved legislatures, should it consider an amendment appropriate. However, in the case of the CVA 2020, the Lords were unlikely to press amendments introducing further safeguards and limitations on powers, including additional review processes, due to the exceptional circumstances of COVID‐19, the time pressures faced, and the upper chamber's respect for the primacy of the Commons.^253^ Importantly, the CVA 2020 furnished the devolved governments with a wide suite of emergency powers, and they were (perhaps understandably) happy to receive them without the limitations and safeguards that might have attended had they been introduced through primary legislation in the more consensual devolved Parliaments.^254^ Furthermore, MLAs, MSs and MSPs generally accepted the CVA 2020's rights‐related impacts as a necessary, if undesirable, side effect of the pandemic response, and welcomed amendments at the Commons introducing a sunset clause and the SMRs,^255^ accepting that they would and could act as safeguards. Only a handful of parliamentarians expressed dissatisfaction with these arrangements, indicating that a shorter time of two or three months between reviews would have been appropriate,^256^ but none pushed the matter or sought to have their government press the central government to strengthen those safeguards.

## CONCLUSION

While these challenges are sharply exposed in the context of the CVA 2020, they are not unique to it. The executive always controls the Commons timetable, MPs’ engagement in the chamber often teeters on the edge of superficiality, and devolved legislatures are always somewhat limited in their ability to influence the central government. However, the extent to which these system features become system failures is in the gift of constitutional actors themselves and, especially, of the executive. The UK constitution has traditionally rested on the assumption that political elites will conduct themselves with self‐restraint^257^ and in recognition of inter‐institutional comity;^258^ that the executive will not take undue advantage of its dominance.

This constitutional mindset is enacted in sometimes quotidian ways: by means of the organisation of parliamentary time, the extent and tone of government engagement with parliamentary committees, and behavioural rituals that serve to recognise government as subordinate to parliamentary will such as Prime Ministerial appearances before the Liaison Committee or making major policy announcements in parliament rather than to the press. In other words, it is now broadly accepted that the constitutional principles of parliamentary sovereignty and accountability to parliament depend to a large extent for their effectiveness on the willingness of the executive to submit to parliament's scrutiny, not to treat parliament as a mere adjunct of the government, and to exercise its power in a manner ‘compatible with the ability of Parliament to carry out its constitutional functions’.^259^ To put it differently, we rely on the proposition that the executive, MPs and devolved governments and legislatures will manifest a constitutionalist mindset (ie a disposition to exercise discretionary power in a manner that respects UK constitutional principles) even when arithmetic, circumstance, and procedure do not strictly require them to.^260^ In particular, this implicates exercising powers in a way that respects parliamentary sovereignty (the ‘bedrock’ of the UK constitution),^261^ parliamentary accountability,^262^ the separation of powers,^263^ the rule of law,^264^ the principle of legality,^265^ and the use of powers in the public – rather than political – interest.

Our narration of the passage and review of the CVA 2020 reveals ways in which such self‐restraint – such constitutionalist mindset – has been lacking in the pandemic, and adds to the emerging political science literature expressing concern about the Johnson Government's apparent ill‐disposition towards accountability.^266^ We contend that decisions as to timing, for example, were not inevitable products of an emergency setting but rather political choices that reflect an executive disposition to act in emergency mode notwithstanding its well‐recognised shortcomings from a constitutional perspective. Even if this can be explained by a sense of pressing circumstance, the wilful decision to create a mode of *ex post facto* review that could not function as compensatory scrutiny (due to its design) and then to ensure that it would not be giving any opportunity to (due to its scheduling) compounds the sense of abandon. In their failure to maximise the limited opportunity the SMR presented, MPs were complicit in this, as to a lesser extent were the devolved legislatures (albeit the constitutional arrangements between central and devolved government present a significant structural challenge in that context). We recognise, of course, that the pandemic raised difficult balancing questions between competing aims. The state had to craft an effective emergency response that enabled the executive to protect public health and the right to life, while also respecting constitutional principles of human rights and parliamentary accountability, and doing so may well result in wide delegation of powers. However, securing both effectiveness in the response and respect for constitutional principle is not a zero‐sum game. Echoing Keith Ewing's salutary reminder, ‘it is Parliament's duty to be at the helm, not only to empower but also to hold government to account’.^267^

This is part of a wider trend of executive dominance in the pandemic context, which goes beyond the CVA 2020 and its review processes. Of particular note is the reliance on delegated legislation in crafting the emergency response at the four nations. While lockdown regulations have been subject to some scrutiny, it has been rare and frustrated further by the procedure used to make this secondary legislation. Key aspects of the emergency response, including measures having wide‐reaching impacts on people's rights and lives, such as lockdown regulations and international travel restrictions, are contained in regulations made under the MAP.^268^ The extensive use of the MAP exacerbates long‐standing concerns about the constitutional implications of delegated law‐making per se.^269^ Parliaments are required to debate and approve these regulations usually weeks after they have come into force, been disseminated, and begun to be complied with by the people and enforced by the police. This over‐reliance on the MAP is consistent with a broader pattern of marginalisation of the legislature that ultimately undermines parliamentary democracy.

We do not suggest this is unique to the United Kingdom; scholars have raised similar concerns in other jurisdictions over the course of the pandemic.^270^ In the UK as well as globally there is growing concern about constitutional decay,^271^ and we contend that our analysis discloses important insights into the functioning of the UK constitution that have resonance beyond the pandemic and within that body of work. We also do not suggest that the current government's apparent ill‐disposition towards constitutionalism finds no precursors in earlier administrations,^272^ even if it may be especially manifest and perhaps unusually acute in this one. Thus, even while narrowly focused, this paper adds to a growing body of literature that should cause us seriously to question the continued commitment of key actors to core constitutional propositions.

While the pandemic has *revealed* key stresses in the practical operation of the constitutional framework in the United Kingdom, it has *not produced* them. Instead, the combination of a large majority in the House of Commons, an emergency/crisis situation requiring complex and urgent state responses, and an executive that seems unwilling to subject itself to the limits imposed by constitutional principle has brought into sharp relief the contrast between constitutional mythology and constitutional reality, between parliamentary accountability and executive dominance.

## Footnotes

1 See for example C. Campbell, *Emergency Law in Ireland 1918‐1925* (Oxford: Clarendon Press, 1994); L. Donohue, *Counter‐Terrorist Law and Emergency Powers in the United Kingdom 1922‐2000* (Dublin: Irish Academic Press, 2001); J. Sim and P. Thomas, ‘The Prevention of Terrorism Act: Normalising the Politics of Repression’ (1983) 10 *Journal of Law and Society* 75; G. Mazarelo, ‘Emergency Legislation in the United Kingdom’ (2009) 11 *European Journal of Law Reform* 323; A. Greene, ‘Questioning executive supremacy in an economic state of emergency’ (2015) 35 *Legal Studies* 594.

2 See for example A. Greene, *Emergency Powers in a Time of Pandemic* (Bristol: Bristol University Press, 2020); S. Thomson and E. Ip, ‘COVID‐19 emergency measures and the impending authoritarian pandemic’ (2020) 7 *Journal of Law and the Biosciences* Isaa064; R. Cormacain, ‘Keeping Covid‐19 emergency legislation socially distant from ordinary legislation: principles for the structure of emergency legislation’ (2020) 8 *The Theory and Practice of Legislation* 245.

3 Coronavirus Act 2020, ss 48 and 49, Sched 18 and 19.

4 K. Fisher, ‘Exploring the temporality in/of British counterterrorism law and law making’ (2013) 6 *Critical Studies on Terrorism* 50.

5 E. Kirton‐Darling, H. Carr and T. Varnava, ‘Legislating for a Pandemic: Exposing the Stateless State’ (2020) 47 *Journal of Law and Society* S302, S306‐S307.

6 HL Deb vol 802 col 1649 24 March 2020 (Lord Bethel).

7 Coronavirus Act 2020, s 98.

8 P. Evans and others (eds), *Parliaments and the Pandemic* (Study of Parliament Group, 2021); P. Dey and J. Murphy, ‘Pandemic Parliamentary Oversight of Delegated Legislation: Comparing the Performance of Westminster Systems’ (2021) 15 *ICL Journal* 465; S. Priddy, ‘Measures taken by the House of Commons in Response to the Covid‐19 Pandemic’ (2021) 1 *International Journal of Parliamentary Studies* 173; S. Chaplin S, ‘Protecting parliamentary democracy in “plague” times: Accountability and democratic institutions during the pandemic’ (2020) 46 *Commonwealth Law Bulletin* 110.

9 For a recent study on role of constitutionalist mindset in the UK, also referred to as ‘self‐restraint’ or the ‘good chap principle’, see A. Blick and P. Hennessey, *Good Chaps No More? Safeguarding the Constitution in Stressful Times* (London: The Constitution Society, 2019). On the idea of comity, see D. Oliver, ‘Parliament and the Courts: A Pragmatic (or Principled) Defence of the Sovereignty of Parliament’ in G. Drewry and A. Horne (eds), *Parliament and the Law* (Oxford: Hart Publishing, 2nd ed, 2018). Critically assessing Johnson's administration, see D. Judge, ‘Walking the Dark Side: Evading Parliamentary Scrutiny’ (2021) 92 *The Political Quarterly* 283.

10 For example see O. Gross, ‘Chaos and Rules’ (2003) 112 *The Yale Law Journal* 1101; D. Dyzenhaus, *The Constitution of Law: Legality in a Time of Emergency* (Cambridge: Cambridge University Press, 2006); T. Sorell ‘Morality and Emergency’ (2002) 103 *Proceedings of the Aristotelian Society* 21; D. Cole, ‘Judging the Next Emergency: Judicial Review and Individual Rights in Times of Crisis’ (2003) 101 *Michigan Law Review* 8; B. Ackerman, *Before the Next Attack: Preserving Civil Liberties in an Age of Terrorism* (New Haven, CT: Yale University Press, 2007); M. Tushnet, ‘The Political Constitution of Emergency Powers’ (2007) 91 *Minnesota Law Review* 1451; N.C. Lazar, *States of Emergency in Liberal Democracies* (Cambridge: Cambridge University Press, 2009) ch 5; J. Ferejohn and P. Pasquino, ‘The Law of the Exception: A Typology of Emergency Powers’ (2004) 2 *International Journal of Constitutional Law* 210; A. Greene, *Permanent States of Emergency and the Rule of Law* (Oxford: Hart Publishing, 2018); K. Loevy, *Emergencies in Public Law: The Legal Politics of Containment* (Cambridge: Cambridge University Press, 2015); E.J. Criddle (ed), *Human Rights in Emergencies* (Cambridge: Cambridge University Press, 2016); M. Head, *Emergency Powers in Theory and Practice: The Long Shadow of Carl* Schmitt (Oxford: Routledge, 2015); O. Gross and F. Ní Aoláin, *Law in Times of Crisis* (Cambridge: Cambridge University Press, 2006).

11 For example see Gross, *ibid*; Ackerman, *ibid*; Tushnet, *ibid*.

12 Dyzenhaus, n 10 above; Sorell, n 10 above; Cole, n 10 above.

13 K. Ewing, ‘Covid‐19: Government by Decree’ (2020) 31 *King's Law Journal* 1, 24.

14
*The Times* 21 April 2021. For an extended version of this letter, see: M. Russell and others, ‘The marginalisation of the House of Commons under Covid has been shocking; a year on, parliament's role must urgently be restored’ *The Constitution Unit Blog* 2021 at https://constitution‐unit.com/2021/04/21/covid‐and‐parliament‐one‐year‐on/ (last visited 30 September 2020). See also R. Cormacain, *Parliamentary Scrutiny of Coronavirus Lockdown Regulations: A Rule of Law Analysis* (London: Bingham Centre, 2020).

15 This is not to suggest that executive dominance is not of broad concern across politico‐legal systems. For a comparative account see for example E. Griglio, ‘Parliamentary oversight under the Covid‐19 emergency: striving against executive dominance’ (2020) 8 *The Theory and Practice of Legislation* 49; P. Guasti, ‘The Impact of the COVID‐19 pandemic in Central and Eastern Europe: The Rise of Autocracy and Democratic Resilience’ (2020) 7 *Democratic Theory* 47.

16 L. Hailsham, *The Dilemma of Democracy: Diagnosis and Prescription* (London: Collins, 1978).

17 A. Lijphart, *Patterns of Democracy: Government Forms and Performance in Thirty‐Six Countries* (New Haven, CT: Yale University Press, 1999).

18 A.C. Eggers and A. Spirling, ‘Ministerial Responsiveness in Westminster Systems: Institutional Choices and House of Commons Debate, 1832‐1915’ (2014) 58 *American Journal of Political Science* 873, 874.

19 M. Loughlin, ‘The Contemporary Crisis of Constitutional Democracy’ (2019) 39 OJLS 435, 439.

20 HC Deb vol 674 cols 35‐101 23 March 2020; HC Deb vol 674 cols 102‐177 23 March 2020; HL Deb vol 802 cols 1649‐1738 24 March 2020; HL Deb vol 802 cols 1739‐1794 25 March 2020.

21 Northern Ireland Assembly Hansard vol 127 No. 5 1‐45 24 March 2020; Scottish Parliament, Official Report, Health and Sport Committee cols 2‐22 24 March 2020; Scottish Parliament, Official Report, Finance and Constitution Committee cols 2‐22 24 March 2020; Scottish Parliament, Official Report cols 54‐76 24 March 2020; Senedd Record cols 252‐365 24 March 2020.

22 HC Deb vol 681 cols 388‐415 30 September 2020; HC Deb vol 691 cols 1109‐1171 25 March 2021; HC Deb vol 701 cols 649‐673 19 October 2021.

23 As Russell and Gover argue, Parliament exerts a subtler and less visible influence in governmental legislation by prompting anticipated reactions on the part of the government (M. Russell and D. Gover, *Legislation at Westminster: Parliamentary Actors and Influence in the Making of British Law* (Oxford: OUP, 2017) 268‐270). Likewise, McHarg identifies a similar effect at Whitehall, as the threat of consent being refused usually leads to negotiation and compromise over the content of UK bills between the central and devolved governments. See A. McHarg, ‘Devolution in Scotland’ in J. Jowell and C. O'Cinneide, *The Changing Constitution* (Oxford: OUP, 9th ed, 2019). Drafters also play an important role in ‘subject[ing] the democratic will to the discipline of reason, in the form of conceptual coherence and respect for established values in the law’ (P. Sales, ‘The Contribution of Legislative Drafting to the Rule of Law’ (2018) 77 CLJ 630, 632).

24 At second reading of the CVA, the Cabinet Secretary for Health went on to say that ‘The Bill is jointly agreed between the four UK Governments.’ (see HC Deb vol 674 col 36 23 March 2020. See also Scottish Parliament, Official Report, col 54 24 March 2020 (Cabinet Secretary for the Constitution, Europe and External Affairs). Mentioning evidence that Scottish civil servants drafted clauses of the CVA 2020, see A. Paun and K. Shuttleworth, ‘Legislating by consent – How to revive the Sewell convention’ *IfG Insight* 2020, 10 at
https://www.instituteforgovernment.org.uk/publications/sewel‐convention (last visited 4 November 2021).

25 Select Committee on the Constitution, *Parliament and the Legislative Process* HL 173‐I (2004) 106‐115; Select Committee on the Constitution, *The Legislative Process: The Passage of Bills Through Parliament* HL 393 (2019) 22‐28.

26 This is especially notable in the context of counter‐terrorism measures. See the analysis in J. Blackbourn, F. de Londras and L. Morgan, *Accountability and review in the Counter‐Terrorist State* (Bristol: Bristol University Press, 2019) ch1.

27 Hansard Society Commission on Parliamentary Scrutiny, *The Challenge for Parliament: Making Government Accountable* (London: Hansard Society, 2001) xi.

28 Russell and others, n 14 above; Judge, n 9 above; Ewing, n 13 above.

29 Notable interventions to date on the Coronavirus Act 2020 include Kirton‐Darling, Carr and Varnava, n 5 above; Ewing, *ibid*; Select Committee on the Constitution, *COVID‐19 and the use and scrutiny of emergency powers* HL 15 (2021). For an account of the Act per se see J. King and N. Byrom, ‘United Kingdom: Legal Response to Covid‐19’ in J. King and O. Ferraz et al (eds), *The Oxford Compendium of National Legal Responses to Covid‐19* (Oxford: OUP, 2021) doi: 10.1093/law‐occ19/e17.013.1 at paras 14 ff.

30 Coronavirus Act 2020, ss 14‐17.

31 Coronavirus Act 2020, Sched 22.

32 Coronavirus Act 2020, ss 37 and 38.

33 Coronavirus Act 2020, ss 59‐70.

34 Coronavirus Act 2020, s 51 and Sched 21.

35 Coronavirus Act 2020, s 10 and Sched 8, 9, 10 and 11.

36 Coronavirus Act 2020, ss 11, 12 and 13.

37 Coronavirus Act 2020, s 23.

38 Coronavirus Act 2020, ss 53‐57.

39 The MAP has been extensively used to introduce so‐called ‘lockdown regulations’ and travel restriction regulations, which in England & Wales have primarily been introduced under powers granted by the Public Health (Control of Disease) Act 1984. In contrast, in Scotland and Northern Ireland, the CVA has been the enabling Act for making ‘lockdown regulations’ under the MAP. For an analysis of the extensive use of the MAP in the Scottish context, see P. Grez Hidalgo, F. de Londras and D. Lock, ‘Use of the Made Affirmative Procedure in Scotland: Reflections from the Pandemic’ (2022) 26 *Edinburgh Law Review* 219. For analysis of the use and extent of the MAP in Westminster see D. Lock, F. de Londras and P. Grez Hidalgo, ‘Delegated Powers in the Pandemic: A Constitutional Bargain Undone?’ (manuscript under review).

40 Coronavirus Act 2020, s 93.

41
*ibid*.

42 The question of whether derogations ought to have been made to the ECHR was the subject of lively scholarly debate. See A. Greene, ‘Derogations, deprivation of liberty and the containment stage of pandemic responses’ (2021) 4 EHRLR 389; A. Greene, ‘On the Value of Derogations from the European Convention on Human Rights in Response to the COVID‐19 Pandemic: A Rejoinder’ (2020) 5 EHRLR 526; K. Dzehtsiarou, ‘Article 15 derogations: Are they really necessary during the COVID‐19 pandemic?’ (2020) 4 EHRLR 359; A. Greene, ‘Derogating from the European Convention on Human Rights in response to the Coronavirus pandemic: if not now, when?’ (2020) 4 EHRLR 262; A. Greene, ‘Derogations, Deprivation of Liberty and the Containment Stage of Pandemic Responses’ (2021) 4 EHRLR 389; M. Scheinin, ‘COVID‐19 Symposium: To Derogate or Not to Derogate’ *Opinio Juris* 2020 at http://opiniojuris.org/2020/04/06/covid‐19‐symposium‐to‐derogate‐or‐not‐to‐derogate/ (last visited 17 October 2021). The list of derogations can be accessed on the Council of Europe website at https://www.coe.int/en/web/conventions/derogations‐covid‐19 (last visited 14 June 2022). For a broader analysis on the use of derogations during the pandemic see for example L. Helfer, ‘Rethinking Derogations from Human Rights Treaties’ (2021) 115 *American Journal of International Law* 20.

43 See for example *Centre for Legal Resources on behalf of Valentin Campeanu* v *Romania* [2014] ECHR 972 at [130]; *Calvelli and Ciglio* v *Italy* [2002] ECHR 3; *Vo* v *France* [2004] ECHR 326; *Lopes de Sousa Fernandes* v *Portugal* [2017] ECHR 1174 at [190]; *Powell* v *United Kingdom* [2000] ECHR 703.

44 International Covenant on Economic, Social and Cultural Rights, Art 12; Committee on Economic, Social and Cultural Rights, ‘General Comment No. 14: The Right to the Highest Attainable Standard of Health (Art. 12)’ (United Nations, Doc. E/C.12/2000/4, 2000).

45 This builds on accountability‐based articulations of review in Blackbourn, de Londras, Morgan, n 26 above.

46 Gross and Ni Aolain, n 10 above, 77‐79; R. Mackenzie‐Gray Scott, ’Rebalancing upstream and downstream scrutiny of government during national emergencies’ UKCLA Blog, 2021 at https://ukconstitutionallaw.org/2021/09/21/richard‐mackenzie‐gray‐scott‐rebalancing‐upstream‐and‐downstream‐scrutiny‐of‐government‐during‐national‐emergencies/ (last visited 28 October 2021).

47 For a general account of the stages of law‐making ‘in the regulatory cycle’ across Europe, see U. Karpen, H. Xanthaki, L. Mader, W. Voermans and R. Cormacain, *Legislation in Europe: A Comprehensive Guide for Scholars and Practitioners* (Oxford: Hart Publishing, 2017) ch 1; R. Thornburn Stern, ‘When the ends justify the means? Quality of law‐making in times of urgency’ (2019) 7 *The Theory and Practice of Legislation* 85 (on refugee‐related law‐making in Sweden).

48 As Thomas Horsley has recently pointed out, ‘… it should fall to Parliament to exercise its legislative supremacy to ensure that the UK constitution discharges its minimum constituting, legitimating and limiting functions.’ See T. Horsley, ‘Constitutional Functions and Institutional Responsibility: a functional analysis of the UK constitution’ (2022) 42 *Legal Studies* 99, 107.

49 Jeff King and Natalie Byrom show that a great deal of the response has been executed using powers under the Public Health (Control of Disease) Act 1984, see King and Byrom, n 29 above, para 22‐24. According to the Hansard Society, ‘Coronavirus Statutory Instruments Dashboard’ *Hansard Society* 2020 at https://www.hansardsociety.org.uk/publications/data/coronavirus‐statutory‐instruments‐dashboard#powers‐used‐by‐ministers (last visited 4 November 2021), Coronavirus‐related Statutory Instruments have been made exercising powers under 134 Acts of Parliament. Furthermore, in Scotland three pieces of primary legislation were passed to frame and enable the Scottish Government's response: the Coronavirus (Scotland) Act 2020, the Coronavirus (Scotland) (No 2) Act 2020 and the Coronavirus (Extension and Expiry) (Scotland) Act 2021. On the constitutionalist implications of relying on multiple legislative frameworks in responding to the pandemic, see Cormacain, n 2 above, 256‐257.

50 This is true, as far as primary legislation is concerned. The four UK government have also made a significant number of regulations under powers granted by the Public Health (Control of Disease) Act 1984 (England & Wales), the Coronavirus Act 2020 and other enabling Acts. We discuss this further in the Conclusion.

51 Hansard Society, n 49 above.

52 Coronavirus Act 2020, ss 88 and 90.

53 On 3 March 2020 the UK Government published its *Coronavirus Action Plan*, which sets out the early emergency response. In Annex A the *Action Plan* outlines to a significant extent the distribution of responsibilities at national level and regional level, and suggests meaningful coordination between the different key institutions including Chief Medical Officers. Department of Health and Social Care, *Coronavirus action plan: a guide to what you can expect across the UK* (2020).

54 Coronavirus Act 2020, Scheds 18 (Northern Ireland) and 19 (Scotland). Importantly, divergence in how pandemic responses and supports are implemented have persisted across the four nations: S. Vicary et al, ‘“It's about how much we can do, and not how little we can get away with”: Coronavirus‐related legislative changes for social care in the United Kingdom’ (2020) 72 *International Journal of Law and Psychiatry* 1.

55 Example of devolved areas: Coronavirus Act 2020, Sched 18 (Northern Ireland), Sched 19 (Scotland). Examples of non‐devolved areas: Coronavirus Act 2020, s 62 (power to postpone a recall petition under the Recall of MPs Act 2015) and Sched 20 (power to suspend port operations).

56 On the facilitative function of the Sewel Convention see for example A. McHarg, ‘Constitutional Change and Territorial Consent: the *Miller* case and the Sewel Convention’ in M. Elliot and others (eds), *The UK Constitution After Miller* (Oxford: Hart Publishing, 2018).

57
*Imperial Tobacco Limited* v *The Lord Advocate* [2012] UKSC 61 at [14] per Lord Hope.

58 N. Burrows, *Devolution* (London: Sweet & Maxwell, 2000) 3.

59 Scotland Act 1998, s 28 (7), Governance of Wales Act 2006, s 107(6); *AXA General Insurance Ltd* v *The Lord Advocate* [2011] UKSC 46 at [46] per Lord Hope; *Reference by the Attorney (The UK Withdrawal from the European Union (Legal Continuity) (Scotland) Bill* [2018] UKSC 64 at [52]; *Reference by the Attorney General and Advocate General for Scotland (United Nations Convention on the Rights of the Child (Incorporation) (Scotland) Bill* [2021] UKSC 42 at [22]‐[36] and [47]‐[55] per Lord Reed.

60 See for example Cabinet Office, *Guide to Making Legislation* (July 2017) 204.

61 House of Commons Reform Committee, *Rebuilding the House* HC 1117 (2009) 22.

62
*ibid*, 162.

63 See n 23 above.

64 As Matt Hancock said at second reading of the CVA: ‘Essentially, the Bill gives all four UK Governments a legislative and regulatory toolkit to respond in the right way at the right time by working through the action plan. While I hope that some of the powers never have to be used, we will not hesitate to act if that is what the situation requires.’ HC Deb vol 674 col 39 23 March 2020. See also Coronavirus Bill Explanatory notes, para 3.

65 Russell and Gover, n 23 above, ch2.

66 Human Rights Act 1998 (Designated Derogation) Order.

67 The Bill was published on 19 September 2001 with the debate commencing on 12 November 2001.

68 ECHR, Art 15. For analysis of whether the UK should have derogated from the ECHR in light of the pandemic see the materials cited in n 42 above.

69 Hybrid procedures were introduced through a series of motions approved after Easter recess, on the 21 and 22 April 2020. See D. Natzel, ‘Coronavirus and the Commons: how the hybrid parliament has enabled MPs to operate remotely’ Constitution Unit, 2020 at https://constitution‐unit.com/2020/05/13/coronavirus‐and‐the‐commons‐how‐the‐hybrid‐parliament‐has‐enabled‐mps‐to‐operate‐remotely/ (last visited 14 June 2022).

70 ‘Prime Minister's statement on coronavirus (COVID‐19): 23 March 2020’ at https://www.gov.uk/government/speeches/pm‐address‐to‐the‐nation‐on‐coronavirus‐23‐march‐2020 (last visited 4 November 2021).

71 See official UK Government data, available at https://coronavirus.data.gov.uk/details/cases (last visited 4 November 2021).

72 HL Deb vol 802 col 1531 19 March 2020.

73 See Standing Orders of the House of Commons No 47; HC Deb vol 674 col 61 (Madam Deputy Speaker reduces interventions to five minutes); col 77 (to four minutes), and col 92 (to three minutes) 23 March 2020.

74 World Health Organisation, *Rational use of personal protective equipment (‎PPE)‎ for coronavirus disease (‎COVID‐19)‎* Interim Guidance (Geneva: WHO, 2020).

75 HC Deb vol 674 col 62 23 March 2020.

76 HC Deb vol 674 col 55 23 March 2020.

77 HC Deb vol 674 col 63 23 March 2020.

78 HL Deb vol 802 col 1775 25 March 2020.

79 HC Deb vol 674 cols 39, 45, 56, 57, 58, 59, 60, 64, 79, 98, 122 23 March 2020; HL Deb vol 802 cols 1651, 1660, 1660, 1660, 1720 24 March 2020; HL Deb vol 802 col 1744, 1744, 1771, 1776, 1777 25 March 2020.

80 HC Deb vol 674 cols 43, 63, 79, 82, 83, 87, 89, 94, 96 23 March 2020; HL Deb vol 802 cols 1656, 1660, 1662, 1667, 1672 24 March 2020.

81 HC Deb vol 674 cols 45, 60, 60, 98, 122, 145 23 March 2020; HL Deb vol 802 cols 1660, 1662, 1708, 1735 24 March 2020; HL Deb vol 802 cols 1775, 1776, 1776 25 March 2020.

82 HC Deb vol 674 cols 84, 88‐89 23 March 2020; HL Deb vol 802, col 1731 24 March 2020.

83 HL Deb vol 802 col 1699 24 March 2020.

84 HC Deb vol 674 cols 57, 58, 79, 89, 98, 122, 23 March 2020; HL Deb vol 802 cols 1708, 1717 24 March 2020; HL Deb vol 802 col 1740 25 March 2020.

85 HC Deb vol 674 col 58‐59 23 March 2020.

86 HC Deb vol 674 col 96 23 March 2020.

87 HC Deb vol 674 cols 95‐96 23 March 2020.

88 HC Deb vol 674 col 56 23 March 2020.

89 HC Deb vol 674 col 59 23 March 2020.

90 HC Deb vol 674 col 88‐89 23 March 2020 (Zarah Sultana MP on immigration detention).

91 HC Deb vol 674 col 91 23 March 2020 (Ruth Jones MP on workers’ rights).

92 We recognise that many of these examples raise complex policy questions and are not ‘solely’ rights‐related issues, but as they do implicate rights their neglect in debates reflects a lack of comprehensive engagement with human rights in the debates.

93 Coronavirus Act 2020, ss 23 and 24.

94 HC Deb vol 674 col 74 23 March 2020 (Robert Largan MP, Conservative).

95 HC Deb vol 674 cols 57, 58, 79, 89, 98, 122 23 March 2020; HL Deb vol 802 cols 1708, 1717 24 March 2020; HL Deb vol 802 col 1740 25 March 2020.

96 Coronavirus Act 2020, ss 14‐17, Sched 12.

97 HL Deb vol 802 col 1777, 1778 25 March 2021.

98 HL Deb vol 802 col 1777, 1778 25 March 2021.

99 HC Deb vol 674 col 45 23 March 2020.

100 Coronavirus Act 2020, s 37 and 38, Sched 16 and 17.

101 HC Deb vol 674 cols 58, 59 23 March 2020.

102 Amendments 68‐71.

103 HC Deb vol 674 cols 84, 88, 89 23 March 2020; HL Deb vol 802 col 1731 24 March 2020. The Government refused to support amendments tabled in response to this concern: NC 7, New Schedule 1.

104 HC Deb vol 674 cols 35, 72, 76, 79, 100 23 March 2020. The government refused to support amendments tabled in response to this concern: NC 35.

105 See for example Select Committee on the Constitution, *COVID‐19 and the use and scrutiny of emergency powers* HL 15 (2021) 216‐217 (on the need to review ‘whether the correct balance was struck between the restrictions on civil liberties and parliamentary scrutiny’ in the pandemic); National Audit Office, *Support for children's education during the early stages of the COVID‐19 pandemic* HC 1239 (2021) 2.7, 2.28 and 2.31 (on the impact of the approach to school closures on children with special education needs, including those with disabilities); Joint Committee on Human Rights, *The Government's response to COVID‐19: human rights implications* HC 265, HL 125 (2020) 138‐142 (on the impact of the pandemic on people in immigration detention) and para 69 (on the disproportionate impact of the pandemic on people from ‘BAME’ communities).

106 HC Deb vol 674 col 39 23 March 2020.

107 Government amendment 52.

108 HC Deb vol 674 col 59 23 March 2020.

109 Government NC 24.

110 HC Deb vol 674 cols 59, 91, 93 23 March 2020.

111 Amendment 13.

112 HL Deb vol 802 col 1778 25 March 2020.

113
*ibid*.

114 The two Scottish Coronavirus Acts incorporated provisions on the advancement of equality and non‐discrimination (see Coronavirus (Scotland) Act 2020, s 9 and Coronavirus (Scotland) (No. 2) Act 2020). The non‐governmental amendment was proposed by a government backbencher, supported by the Scottish Government and approved without a division (Scottish Parliament Official Record 19 May 2020 cols 19 and 20). There is also a resemblance between these provisions and Sched 5, part 5 of the Terrorism Prevention and Investigation Measures 2011, which provides that ‘nothing … in the rules of court’ governing legal proceedings reviewing the imposition of Terrorism Prevention and Investigation Measures ‘is to be read as requiring the relevant court to act in a manner inconsistent’ with ECHR, Art 6.

115 HC Deb vol 674 col 102 24 March 2020.

116 NC 15 (‘Emergency arrangements concerning medical practitioners’); NC 16 (‘Disapplication of limit under section 8 of the Industrial Development Act 1982’); NC 17 (‘Elections and referendums due to be held in England in period after 15 March 2020’); NC 18 (‘Elections due to be held in Wales in period after 15 March 2020’); NC 19 (‘Six‐monthly parliamentary review’); NC 20 (‘Local authority meetings’); NC 21 (‘Extension of BID arrangements: England’); NC 22 (‘Extension of BID arrangements: Northern Ireland’); NC 23 (‘Extension of time limits for retention of fingerprints and DNA profiles’); NC 24 (‘Residential tenancies: protection from eviction’); NC 25 (‘HMRC functions’); NC 26 (‘Up‐rating of working tax credit etc’); NC 30 (‘Business tenancies in England and Wales: protection from forfeiture etc’); NC 31 (‘Business tenancies in Northern Ireland: protection from forfeiture etc’); NC 1 (‘Postponement of General Synod elections’); New Schedule 3 (’Residential tenancies: protection from eviction’), Schedule 7 (‘Mental Health: England and Wales’), Schedule 27 (‘Deceased wishes’), Schedule 18 (‘Health protection regulations: Scotland’), Schedule 20 (‘powers relating to potentially infectious persons’), Schedule 21 (‘powers to issue directions relating to events, gatherings and premises’), Schedule 23 (‘live links in criminal hearings related to extradition’), Schedule 25 (‘cross‐reference to rights to appeal to a magistrate court against requirements or restrictions’).

117 HC Deb vol 674 col 102 24 March 2020.

118 Provisional grouping and selection of Amendment by Chairman of Ways and Means, Coronavirus Bill at
https://publications.parliament.uk/pa/bills/cbill/58‐01/0122/amend/coronavirusfinalselection.pdf (last visited 4 November 2021).

119 Amendments 1, 5, 6, 7 and 13.

120 Amendments 2, 3, 4, 8, 9, 10, 11, 12 and 14.

121 M. Russell and P. Cowley, ‘The Policy Power of the Westminster Parliament: The “Parliamentary State” and the Empirical Evidence’ (2017) 29 *Governance: An International Journal of Policy, Administration, and Institutions* 121; Russell and Gover, n 23 above, ch 2.

122 Notice of amendments 19 March 2020, New Clause 13, New Clause 33, New Clause 34.

123 NC 19.

124 HC Deb vol 674 col 79 23 March 2020.

125 NC 24.

126 For example, HC Deb vol 674 col 59 23 March 2020.

127 World Health Organisation, ‘WHO Director‐General's opening remarks at the media briefing on COVID‐19 ‐ 11 March 2020’ at https://www.who.int/dg/speeches/detail/who‐director‐general‐s‐opening‐remarks‐at‐the‐media‐briefing‐on‐covid‐19‐11‐march‐2020 (last visited 1 October 2020).

128 Only in Scotland were regulations imposing the first lockdown made under powers provided by the CVA (see introductory text of The Health Protection (Coronavirus) (Restrictions) (Scotland) Regulations 2020). While this might suggest that Scottish Ministers lacked pre‐existing powers to impose lockdown regulations, the Scottish Minister for Health argued that the Public Health etc (Scotland) Act 2008 already provided most of the powers granted by the CVA, but that the CVA had been used to prevent discussions about the extent and nature of those powers, and to have a coherent four‐nations approach (see Scottish Parliament, Official Report, Health and Sports Committee cols 10‐11 24 March 2020). In contrast, the first lockdown in England and in Wales, was imposed under powers provided by the Public Health Protection (Control of Disease) Act 1984 (see introductory texts to The Health Protection (Coronavirus, Restrictions) (England) Regulations 2020, and to The Health Protection (Coronavirus Restrictions) (Wales) Regulations 2020). Lockdown in Northern Ireland was imposed under powers provided in the Public Health Act (Northern Ireland) 1967 (see introductory text to The Health Protection (Coronavirus, Restrictions) Regulations (Northern Ireland) 2020).

129 HC Deb vol 674 cols 85‐86 (Adam Afriyie MP, Conservative), 117 (Chris Bryant MP, Labour; Andrew Mitchell MP, Conservative), 118 (David Davis MP, Conservative) 23 March 2020. For a critical view of the decision enact the CVA instead of employing the Civil Contingencies Act 2004, see A. Blick and C. Walker, ‘Why did government not use the Civil Contingencies Act?’ *The Law Society Gazette* 2 April 2020 at https://www.lawgazette.co.uk/legal‐updates/why‐did‐government‐not‐use‐the‐civil‐contingencies‐act/5103742.article (last visited 1 November 2021). See also the critique by Ewing, n 13 above, 5, 12‐16, and Select Committee on the Constitution, *COVID‐19 and the use and scrutiny of emergency powers* HL 15 (2021) 26‐42

130 HL Deb vol 802 col 1776 24 March 2020.

131 In Scotland these include subject committees and a more proportionate electoral system (C. Carman and M. Shephard, ‘Committees in the Scottish Parliament’ in C. Jeffery and J. Mitchell (eds), *The Scottish Parliament 1999–2009: The First Decade* (Edinburgh: Luath Press, 2009) 25; E. St Denny, ‘The Scottish Parliament’ in M. Keating (ed), *The Oxford Handbook of Scottish Politics* (Oxford: OUP, 2020) 482). Wales pursued a deliberate design to resemble Westminster ‘as little as possible’ (A. Trench, ‘Wales and the Westminster Model’ (2010) 63 *Parliamentary Affairs* 117, 118), while in Northern Ireland the Assembly is designed according to a consociational, power‐sharing design with multi‐party legislative scrutiny (B. Dickson, ‘Devolution in Northern Ireland’ in J. Jowell and C. O'Cinneide (eds), *The Changing Constitution* (Oxford: OUP, 9th ed, 2019)).

132 Dickson, *ibid*, 249‐254.

133
https://ballotbox.scot/sp21#overall. By the end of August 2021, the Scottish Government announced an agreement for a governing partnership with the Greens. See https://www.gov.scot/publications/agreement‐scottish‐green‐party‐first‐ministers‐statement‐31‐august‐2021/ (last visited 4 November 2021).

134 Trench, n 131 above, 126.

135 J. McGarry and B. O'Leary, ‘Introduction: Consociational Theory and Northern Ireland’ in J. McGarry and B. O'Leary (eds), *The Northern Ireland Conflict: Consociational Engagements* (Oxford: OUP, 2004) 29‐30.

136 As the Scottish Cabinet Secretary for the Constitution put it in the LCM debate: ‘The bill is the result of a great deal of intensive work between the UK Government and the devolved Administration.’ (Scottish Parliament, Official Report col 54 24 March 2020).

137 For instance, Coronavirus Act 2020, ss 48 and 49, Sched 18 and 19, s 51, Sched 19, s 52, Sched 22.

138 Consider for example this representative intervention by Justin McNulty MLA (SDLP): ‘In an ideal world, we would have weeks and months to scrutinise a Bill such as this. That is not possible because we are on the brink of an extraordinary crisis. The measures proposed are harsh but necessary. Coronavirus/COVID‐19 is a global force majeure.’ (Northern Ireland Assembly Hansard, vol 127, No 5, 27 24 March 2020).

139 Select Committee on the Constitution, ‘Parliament’ n 25 above, 117; ‘The Legislative Process’ n 25 above, 40.

140 In Northern Ireland the motion was tabled five days in advance (on 19 March 2020), in Scotland it was tabled only one day in advance (23 March 2020), and in Wales on the very same day of the debate (24 March 2020).

141 It is difficult to draw comparisons between the duration of these LCMs and others. LCM debates are not usually contentious, and until 2018 legislative consent was withheld on only nine occasions out of 200 opportunities (once in Northern Ireland and Scotland, and seven times in Wales). See Paun and Shuttleworth, n 24 above, 12. This has changed somewhat due to Brexit. Between 2018 and May 2020, the House of Commons Library identifies 13 new cases where consent has been withheld. See G. Cowie and D. Torrance, *Devolution: The Sewel Convention* Briefing Paper CBP‐8883 (House of Commons Library, 2020).

142 To abide by the Scottish Government's social distancing guidelines, the Finance and Constitution Committee agreed to limit attendance to the oral evidence session to five members, selected on a cross‐party basis. See Scottish Parliament, Official Report, Finance and Constitution Committee col 1 24 March 2020.

143 In Wales, this included two statements by the First Minister of Wales and the Minister for Health and Social Services, as well as a motion to suspend standing orders due to Coronavirus. See Agenda at https://business.senedd.wales/documents/g6243/Agenda%20Tuesday%2024‐Mar‐2020%2010.00%20Plenary%20‐%20Fifth%20Senedd.pdf?T=0 (last visited 1 November 2020). In Scotland, on the other hand, there were 20 items on the agenda, including COVID‐19 related statements by the First Minister of Scotland, the Cabinet Secretary for Health and Sport, and the Cabinet Secretary for Justice. See Business Motion S5M‐21332, available at https://www.parliament.scot/chamber‐and‐committees/votes‐and‐motions/votes‐and‐motions‐search/S5M‐21332 (last visited 4 November 2021).

144 There are no records of why this amendment to the government motion was not debated. Considering the nature of the amendment, it might be that the Speaker of the Northern Ireland Assembly was of the view that the amendment was beyond the legislative competence of the Assembly. The amendment, promoted by Gerry Caroll MLA, concerned an attempt to amend clause 82 of the Coronavirus Bill, incorporating a vote at the Welsh Senedd on the continuation of the powers every two months. The text of this amendment is available at
http://aims.niassembly.gov.uk/plenary/details.aspx?tb=&tbv=0&tbt=&pt=&ptv=0&ptt=&mc=&mcv=0&mct=&mt=&mtv=0&mtt=&sp=1&spv=0&spt=&ss=&tm=&per=1&fd=24/03/2020&td=24/03/2020&tit=&txt=&pm=&it=0&pid=3&sid=p&doc=305593&pn=0&ba=1&sd=0&se=.

145 Senedd Record col 291 24 March 2020 (Helen Mary Jones SM, Plaid Cymru).

146 Northern Ireland Hansard vol 127 No 5 8 24 March 2020 (John O'Dowd MLA, Sinn Fein).

147 Scottish Parliament, Official Report cols 63 (Patrick Harvie MSP, Green), referred to the Scottish Children and Young People's Commissioner who had raised concerns about the emergency measures, as well as other disability and care organisations); 64 and 65 (Willie Rennie MSP (Liberal Democrat) and Joan McAlpine MSP (SNP) echoed concerns raised by Inclusion Scotland regarding measures that increase flexibility for social care decision‐making by local authorities); 61 (Alex Rowley MSP, Labour), raised concerns about access to PPE for front line health and social care workers, which in turn had been made to him by Joint Trade Unions in Fife) 24 March 2020.

148 Senedd Record col 290 24 March 2020 (Helen Mary Jones SM, Plaid Cymru); Northern Ireland Assembly Hansard vol 127 No 5 37 (Jonathan Buckley MLA, DUP), 33‐34 (Jim Allister MLA, Traditional Unionist Voice) 24 March 2020.

149 In Scotland, the Finance and Constitution Committee and the Health and Sport Committee held oral evidence sessions. In Northern Ireland, the Executive provided an advance briefing to the chairs of the main parliamentary committees, and each chair then had the chance to speak on behalf of their committees in the Assembly debate on the LCM. Similarly, the Welsh Minister for Health and Social Services appeared before the Health, Social Care and Sport Committee to brief committee members on the, at the time, imminent passage of the CVA (Senedd Record, Health, Social Care and Sport Committee cols 162‐330 18 March 2020).

150 As the Delegated Powers and Law Reform Committee had a closed session on the LCM there are no public records of this meeting.

151 Scottish Parliament, Official Report, Finance and Constitution Committee cols 9, 12‐14, 17‐18 24 March 2020.

152 Scottish Parliament, Official Report, Health and Sport Committee cols 6‐10, 11, 12‐16, 17 24 March 2020.

153 Scottish Parliament, Official Report cols 56‐59 24 March 2020.

154 There are exceptions: Adam Tomkins MSP (Conservative) made a developed argument about the significance of human rights law in the pandemic context and the tests applicable to assess the legitimacy of the measures (Scottish Parliament, Official Report cols 71‐73 24 March 2020); Martina Anderson MLA (Sinn Fein) stressed the significance of international human rights law in considering proportionality and necessity, (Northern Ireland Assembly Hansard vol 127 No 5 25‐26 24 March 2020), and Rachel Woods MLA (Green) emphasised the importance of ensuring that emergency powers are exercised in a rights‐compliant way (Northern Ireland Assembly Hansard, vol 127 No 5 29‐31 24 March 2020).

155 For instance, consider Jim Allister MLA (TUV)’s intervention, for whom ‘these are proposals that none of us, as legislatures, should be at ease with, because they strip out our rights and protections that all of us should value.’ (Northern Ireland Assembly Hansard vol 127 No 5 32 24 March 2020). See also Senedd Record col 304 24 March 2020 (Caroline Jones MS, Independent); Scottish Parliament, Official Report cols 66‐67 24 March 2020 (Ruth Davidson MSP, Conservative).

156 Northern Ireland Assembly Hansard vol 127 No 5 23‐24 (Mike Nesbitt MLA, UUP); 25‐26 (Martina Anderson MLA, Sinn Fein); 29‐31 (Rachel Woods MLA, Green) 24 March 2020.

157 For instance, see Senedd Record cols 275 (Mick Antoniw MS, Lab, Chair of the Senedd Legislation, Justice and Constitution Committee), 281 (Paul Davies MS, Conservative), 304 (Caroline Jones MS, Independent) 24 March 2020. For the Scottish Parliament, see Scottish Parliament, Official Report cols 66‐67 24 March 2020 (Joan McAlpine MSP, SNP and Ruth Davidson MSP, Conservative). For the Northern Ireland Assembly, see Northern Ireland Assembly Hansard vol 127 No 5 6 (Pam Cameron MLA, DUP, Deputy Chairperson of the Committee for Health) 25‐26 (Martina Anderson MLA, Sinn Fein); 28 (Matthew O'Toole MLA, SDLP) 24 March 2020.

158 See the discussion of asylum seekers’ rights by Patrick Harvie MSP, Green (Scottish Parliament, Official Report, Finance and Constitution Committee col 13 24 March 2020. See also his intervention at the Chamber: Scottish Parliament, Official Report col 63 24 March 2020) and response by the Cabinet Secretary for the Constitution (Scottish Parliament, Official Report, Finance and Constitution Committee col 13 24 March 2020), and the discussion of detainees’ needs by Patsy McGlone MLA (SDLP) (Northern Ireland Assembly Hansard vol 127 No 5 22 24 March 2020).

159 Northern Ireland Assembly Hansard vol 127 No 5 35 24 March 2020 (Gerry Carroll MLA, People Before Profit); Scottish Parliament, Official Report col 62 24 March 2020 (Patrick Harvie MSP, Green).

160 Scottish Parliament, Official Record cols 66 (Joan Mc Alpine MSP, SNP) and 72 (Adam Tomkins MSP, Conservative) 24 March 2020; Northern Ireland Assembly Hansard vol 127 No 5 25‐26 (Martina Anderson MLA, Sinn Fein), 29‐31 (Rachel Woods MLA, Green) 24 March 2020.

161 Senedd Record cols 275 (Mick Antoniw MS, Labour), 281 (Paul Davies MS, Conservative), 304 (Caroline Jones MS, Independent), 339 (Neil McEvoy MS, Propel) 24 March 2020; Scottish Parliament, Official Record col 59 (Murdo Fraser MSP, Conservative), 67 (Ruth Davidson MSP, Conservative), 69 (Monica Lennon MSP, Labour) 24 March 2020; Northern Ireland Hansard, vol 127 No 5 12 (Steve Aiken MLA, UUP) and 28 (Matthew O'Toole MLA, SDLP) 24 March 2020.

162 See Department for Health, Northern Ireland Government, Draft Legislative Consent Memorandum – Coronavirus Bill, 19 March 2020, 17, which only contains a pro‐forma statement of compliance with human rights and equality. Neither the Scottish nor the Welsh memorandums engage with human rights at all. See Scottish Government, ‘Legislative Consent Memorandum – Coronavirus Bill’ (2020) at https://archive2021.parliament.scot/S5_Bills/SPLCM‐S05‐36.pdf (last visited 4 November 2021); Welsh Government, ‘Legislative Consent Memorandum – Coronavirus Bill’ (2020) at https://senedd.wales/laid%20documents/lcm‐ld13106/lcm‐ld13106%20‐e.pdf (last visited 4 November 2021).

163 Northern Ireland Assembly Hansard vol 127 No 5 28 24 March 2020 (Justin McNulty MLA, SDLP); Scottish Parliament, Official Record col 63 (Patrick Harvie MSP, Green) and 66 (Joan McAlpine MSP, SNP) 24 March 2020; Scottish Parliament, Official Record, Finance and Constitution Committee col 18 (Angela Constance MSP, SNP) and col 66 (Joan McAlpine MSP, SNP) 24 March 2020; Senedd Record col 290 24 March 2020 (Helen Mary Jones MS, Plaid Cymru).

164 Paula Bradshaw MLA, Alliance Party (Northern Ireland Assembly Hansard vol 127 No 5 13‐14 24 March 2020); Rachel Woods MLA, Green (Northern Ireland Assembly Hansard vol 127 No 5 30 24 March 2020); Sandra White MSP, SNP (Scottish Parliament, Official Report, Health and Social Care Committee col 9 24 March 2020); Angela Constance MSP, SNP (Scottish Parliament, Official Report, Finance and Constitution Committee col 16 24 March 2020); Alex Cole‐Hamilton MSP, Liberal Democrat (Scottish Parliament, Official Report, Health and Social Care Committee cols 6‐7 24 March 2020); and Anas Sarwar MSP, Labour (Scottish Parliament, Official Report col 69 24 March 2020).

165 Northern Ireland Assembly Hansard vol 127 No 5 4 24 March 2020.

166 Scottish Parliament, Official Report, Health and Social Care Committee cols 9‐10 24 March 2020.

167 Scottish Parliament Official Report col 59‐60 24 March 2020.

168 Scottish Parliament, Official Report, Finance and Constitution Committee col 14 24 March 2020; Scottish Parliament, Official Report col 54 24 March 2020.

169 Coronavirus (Scotland) Act 2020, ss 12 and 15; Coronavirus (Scotland) (No 2) Act 2020, ss 9 and 12.

170 Scottish Parliament, Official Report, Finance and Constitution Committee cols 12‐13 24 March 2020.

171 Coronavirus (Scotland) Act 2020, s 2 and Sched 1; Coronavirus (Scotland) (No 2) Act 2020, s 1 and Sched 1, Parts 1 and 2.

172 See Senedd Record cols 276‐278 (Mick Antoniw MS, who suggested a Welsh review), 290 (Helen Mary Jones MS, Plaid Cymru). 311‐312 (Alun Davies MS, Labour), 323 (Sian Gwenllian MS, Plaid Cymru) 24 March 2020.

173 Senedd Record col 346 24 March 2020.

174 Reporting practices in Scotland have been regular and supported by specific provisions in the Scottish Coronavirus Acts. So far, the Scottish Government has published thirteen reports. The reports are available at https://www.gov.scot/collections/coronavirus‐covid‐19‐legislation/ (last visited 26 June 2022). In contrast, the Welsh Government only made a political commitment to regular reporting. In practice, the Welsh Government has published four reports. Which are available at https://gov.wales/coronavirus‐pandemic‐legislation‐reports‐senedd‐cymru (last visited 26 June 2022).

175 HC Deb vol 674 col 36 23 March 2020 (Matt Hancock, Conservative).

176 D. Lock, P. Grez Hidalgo and F. de Londras, ‘Parliament's One‐Year Review of the Coronavirus Act 2020: Another Example of Parliament's Marginalisation in the Covid‐19 Pandemic’ (2021) 92 *The Political Quarterly* 699.

177 HC Deb vol 674 col 70 23 March 2020 (David Davis, Conservative), HC Deb vol 674 col 38 23 March 2020 (Andrew Mitchell, Conservative), HC Deb vol 681 col 403 30 September 2020 (Ian Blackford, SNP), HC Deb vol 691 col 1124 25 March 2021 (Martyn Day, SNP), HC Deb vol 701 col 662 19 October 2021 (Martyn Day, SNP).

178 HC Deb vol 681 col 403 30 September 2020 (Ian Blackford, SNP), HC Deb vol 691 col 1124 25 March 2021 (Martyn Day, SNP), HC Deb vol 701 col 662 19 October 2021 (Martyn Day, SNP).

179 HC Deb vol 681 col 392 30 September 2020.

180 HC Deb vol 681 col 393 30 September 2020.

181 The SMR3 took place on 19 October 2021.

182 HC Deb vol 701 col 651 19 October 2021.

183 HC Deb vol 691 col 1135 25 March 2021.

184 HC Deb vol 691 col 1115 25 March 2021 (Matt Hancock, Conservative).

185 The one‐year report on the status of the non‐devolved provisions of the Coronavirus Act, Health Protection (Coronavirus, Restrictions) (Steps) (England) Regulations 2021, and a motion to extend the temporary Standing Orders governing MP's proxy voting and virtual participation rights during the pandemic (SI 2021/234).

186 These were to govern the next phase of ‘lockdown’ and introduced, among other things, extensive restrictions on travelling abroad including creating a criminal offence of merely travelling to a port or airport in anticipation of non‐compliant foreign travel (Health Protection (Coronavirus, Restrictions) (Steps) (England) Regulations 2021, s 10).

187 Notably, even if there had only been one motion, the allocated time may still have been insufficient; as Karen Bradley MP (Conservative) put it, ‘three‐and‐a‐half hours for a debate on these topics is simply not enough’, noting that the ‘length of the call list makes it easy to see just how many Members wish to take part’. HC Deb vol 691 col 1128 25 March 2021.

188 Highlighted by the Select Committee on the Constitution, *COVID‐19 and Parliament* HL 4 (2021) 59‐61.

189 HC Deb vol 681 col 411 30 September 2020.

190 HC Deb vol 701 col 650 19 October 2021.

191 HC Deb vol 701 col 666 19 October 2021.

192 The annual report on the CVA, published just before the one year review stated that regulations made under CVA, s 24 would soon be expired. This section provides the Government with powers to extend time limits on the retention of fingerprints and DNA profiles being held in the interest of national security. This was welcomed by Steve Brine MP (Conservative) in the only reference to that report during the SMR2 (HC Deb vol 691 col 1137 5 March 2021). At the time the Government did not respond on this point, but on 19 April 2021, while Parliament was in recess, the Government published corrections to the report (Corrections made to the One Year Report on the status on the non‐devolved provisions of the Coronavirus Act 2020 (19 April 2021) at https://assets.publishing.service.gov.uk/government/uploads/system/uploads/attachment_data/file/978843/corrections‐to‐coronavirus‐act‐one‐year‐status‐report.pdf (last visited 3 November 2021). These stated that, contrary to what the original report had claimed, only one of two regulations made under CVA, s 24 would actually be expired. This meant that the Coronavirus (Retention of Fingerprints and DNA Profiles in the Interests of National Security) (No 2) Regulations 2020 would continue to operate.

193 See House of Commons Library, *Renewing parts of the Coronavirus Act, new public health regulations and parliamentary proceedings* CBP 9180 (2021); House of Commons Library, *The renewal of temporary provisions under the Coronavirus Act 2020* CBP 9342 (2021).

194 HC Deb vol 691 col 1116 25 March 2021 (Mark Harper, Conservative) – repeated this reference in HC Deb vol 691 col 1145 25 March 2021, HC Deb vol 691 col 1137 25 March 2021 (Steve Baker, Conservative).

195 Joint Committee on Human Rights, *The Government's response to Covid‐19: human rights implications* HC 265 (2020) (HC Deb vol 691 col 1120 25 March 2021, Jonathan Ashworth MP, Labour); Procedure Committee, *Back to the Future? Procedure under coronavirus restri*ctions HC 1282 (2021) (in HC Deb vol 691 col 1138 25 March 2021, Owen Thompson MP, SNP), Public Administration and Constitutional Affairs Committee, *Government transparency and accountability during Covid 19: The data underpinning decisions* HC 803 (2021) (HC Deb vol 691 col 1146 25 March 2021, Rachel Hopkins MP, Labour); Women and Equalities Committee, *Unequal impact? Coronavirus, disability and access to services* HC 1050 (2020) (HC Deb vol 691 cols 1158‐1159 25 March 2021, Caroline Nokes MP, Conservative).

196 Jonathan Ashworth MP (Labour) (HC Deb vol 701 col 658 19 October 2021), Dawn Butler MP (Labour) (HC Deb vol 701 col 665 19 October 2021) and David Davis MP (Conservative) (HC Deb vol 701 col 666 19 October 2021) referred to a report published by the Health and Social Care, and Science and Technology Committees shortly before the SMR3, which was critical of the Government's pandemic response: Health and Social Care and Science and Technology Committees, *Coronavirus: lessons learned to date* HC 92 (2021).

197 A full list of parliamentary committee publications related to COVID‐19, and the date of their publication can be found at https://blog.bham.ac.uk/cvro/covid‐19‐reviews/committee‐inquiries‐westminster/.

198 For example, Comptroller and Auditor General, *Overview of the UK government's response to the COVID‐19 pandemic* HC 366 (2020), *Readying the NHS and adult social case in England for COVID‐19* HC 367 (2020), *Investigation into how government increased the number of ventilators available to the NHS in response to COVID‐19* HC 731 (2020), *Implementing employment support schemes in response to the COVID‐19 pandemic* HC 862 (2020), *The supply of personal protective equipment (PPE) during the COVID‐19 pandemic* HC 961 (2020), *Investigation into government procurement during the COVID‐19 pandemic* HC 959 (2020), *Investigation into the free school meals voucher scheme* HC 1036 (2020), *Investigation into the housing of rough sleepers during the COVID‐19 pandemic* HC 1075 (2021), *Protecting and supporting the clinically extremely vulnerable during the lockdown* HC 1131 (2021), *Local government finance in the pandemic* HC 1240 (2021).

199 Equality and Human Rights Commission, *How coronavirus has affected equality and human rights* (2020).

200 For example, see Office of National Statistics, ‘Early assessment of the impact of the coronavirus pandemic on the UK's financial accounts’ 3 July 2020 at https://www.ons.gov.uk/economy/nationalaccounts/uksectoraccounts/articles/earlyassessmentoftheimpactofthecoronaviruspandemicontheuksfinancialaccounts/2020‐07‐03 (last visited 5 November 2021); ‘Impact of coronavirus in care homes in England: 26 May to 19 June 2020’ 3 July 2020 at https://www.ons.gov.uk/peoplepopulationandcommunity/healthandsocialcare/conditionsanddiseases/articles/impactofcoronavirusincarehomesinenglandvivaldi/26mayto19june2020 (last visited 5 November 2021); ‘Coronavirus and the impact on caring’ 9 July 2020 at https://www.ons.gov.uk/peoplepopulationandcommunity/healthandsocialcare/conditionsanddiseases/articles/morepeoplehavebeenhelpingothersoutsidetheirhouseholdthroughthecoronaviruscovid19lockdown/2020‐07‐09 (last visited 5 November 2021); ‘Coronavirus and homeschooling in Great Britain: April to June 2020’ 22 July 2020 at https://www.ons.gov.uk/peoplepopulationandcommunity/educationandchildcare/articles/coronavirusandhomeschoolingingreatbritain/apriltojune2020 (last visited 5 November 2021); ‘Parenting in lockdown: Coronavirus and the effects on work‐life balance’ 22 July 2020 at https://www.ons.gov.uk/peoplepopulationandcommunity/healthandsocialcare/conditionsanddiseases/datasets/parentinginlockdowncoronavirusandtheeffectsonworklifebalance (last visited 5 November 2021).

201 In SMR1, two NGO reports were mentioned: Inclusion London, ‘Abandoned, forgotten and ignored – the impact of Covid‐19 on Disabled people’ Interim Report, June 2020 at https://www.inclusionlondon.org.uk/wp‐content/uploads/2020/06/Abandoned‐Forgotten‐and‐Ignored‐Final‐1.pdf (last visited 4 November 2021) (HC Deb vol 681 col 410 25 September 2020); BMJ, ‘Covid‐19: excess all cause mortality in domiciliary care’ 2020 at https://doi.org/10.1136/bmj.m2751 (last visited 3 November 2021) (HC Deb vol 681 col 407 25 September 2020). Other prominent important accounts of human rights impacts were not mentioned. For example, see Amnesty International, ‘Coronavirus: the effect on human rights impact in the UK’ 24 April 2020 at https://www.amnesty.org.uk/human‐rights‐uk/coronavirus‐effect‐human‐rights (last visited 4 November 2021), The Children's Society, ‘Life on Hold: children's well‐being and Covid‐19’ 2020 at https://www.childrenssociety.org.uk/information/professionals/resources/life‐on‐hold (last visited 4 November 2021).

202 Joint Committee on Human Rights, n 195 above.

203 The relevant provisions expired are set out in the following reports Department for Health and Social Care, *Two monthly report on the status on the non‐devolved provisions of the Coronavirus Act 2020* CP 282 (2020), Department for Health and Social Care, *Coronavirus Act one‐year reports: March 2021 (Amended)* (2021); Department for Health and Social Care, *Ninth two‐monthly report on the Coronavirus Act 2020* CP 534 (2021).

204 HC Deb vol 681 cols 390, 399 (4 mentions), 400, 403, 410 (7 mentions) and 412 30 September 2020.

205 HC Deb vol 691 cols 1124, 1129 (2 mentions), 1131 (2 mentions), 1135 (2 mentions), 1142, 1152 (2 mentions), 1162 25 March 2021.

206 HC Deb vol 701 col 656, 662, 665, 668 19 October 2021.

207 For example, B. Etheridge and L. Spantig, ‘The Gender Gap in Mental Well‐Being During the Covid‐10 Outbreak: Evidence from the UK’ Institute for Social and Economic Research, University of Essex, Working Paper No 2020‐08, 2020.

208 For example, S. Davidge, ‘A Perfect Storm: The Impact of the Covid‐19 Pandemic on Domestic Abuse Survivors and the Services Supporting Them’ *Women's Aid* August 2020 at https://www.womensaid.org.uk/wp‐content/uploads/2020/08/A‐Perfect‐Storm‐August‐2020‐1.pdf (last visited 4 November 2021).

209 Housing, Communities and Local Government Committee, *Protecting rough sleepers and renters: Interim Report* HC 309 (2020).

210 Rethink Mental Illness, ‘People living with severe mental illness at heightened risk of relapse or crisis during pandemic’ 4 June 2020 at https://www.rethink.org/news‐and‐stories/news/2020/06/people‐living‐with‐severe‐mental‐illness‐at‐heightened‐risk‐of‐relapse‐or‐crisis‐during‐pandemic/ (last visited 25 October 2021).

211 M. Davies, ‘Covid‐19: how is it impacting on prisoners’ health?’ (Nuffield Trust Comment, 2020) at https://www.nuffieldtrust.org.uk/news‐item/covid‐19‐how‐is‐it‐impacting‐on‐prisoners‐health#easing‐lockdown‐in‐prisons (last visited 25 October 2021).

212 Joint Committee on Human Rights, n 189 above at 137‐140.

213 M. Busby and M. Gidda, ‘BAME people fined more than white population under coronavirus laws’ *The Guardian* 26 May 2020) at https://www.theguardian.com/world/2020/may/26/bame‐people‐fined‐more‐than‐white‐population‐under‐coronavirus‐laws (last visited 25 October 2021). Human rights issues that were raised included the right to care for disabled people (HC Deb vol 681 cols 390, 399, 399, 410 30 September 2020), workers’ rights (HC Deb vol 681 col 400 30 September 2020) wrongful prosecutions and the detention of ‘potentially infectious’ persons under Schedule 21 of the Act (HC Deb vol 681 cols 399, 400, 411 30 September 2020), higher COVID‐19‐related deaths among BAME populations and disabled people and in care homes (HC Deb vol 681 cols 397‐399, 406, 407 30 September 2020), and the impact of the CVA education measures on children with special educational needs) and from economically disadvantaged backgrounds (HC Deb vol 681 cols 398, 399, 410 30 September 2020).

214 The key human rights issues raised related to the lack of explicit exemptions for protests in the second lockdown regulations (HC Deb vol 691 cols 1121, 1125, 1129, 1148, 1161, 1162 25 March 2021), and wrongful prosecutions and the detention of ‘potentially infectious’ persons (HC Deb vol 691 cols 1120, 1124, 1130, 1150, 1151 25 March 2021).

215 A. Topping and N. McIntyre, ‘UK school closures mean mothers will take twice as much unpaid leave as fathers’ *The Guardian* 8 January 2021 at https://www.theguardian.com/education/2021/jan/08/uk‐covid‐school‐closures‐mean‐mothers‐will‐take‐twice‐as‐much‐unpaid‐leave‐as‐fathers‐poll (last visited 27 October 2021).

216 E. Romanou and E. Belton, ‘Isolated and struggling: social isolation and the risk of child maltreatment, in lockdown and beyond’ NSPCC Evidence team, 2020 at https://learning.nspcc.org.uk/media/2246/isolated‐and‐struggling‐social‐isolation‐risk‐child‐maltreatment‐lockdown‐and‐beyond.pdf (last visited 4 November 2021). I. Pollock, ‘Covid: Child abuse referrals up nearly 80%, says NSPCC’ *BBC News* 18 December 2020 at https://www.bbc.co.uk/news/uk‐wales‐55352968 (last visited 25 October 2021).

217 S. Minson, ‘The impact of COVID‐19 prison lockdowns on children with a parent in prison’ Centre for Criminology, University of Oxford, 2021 at https://www.law.ox.ac.uk/sites/files/oxlaw/the_impact_of_covid‐19_prison_lockdowns_on_children_with_a_parent_in_prison.pdf (last visited 27 October 2021).

218 Comptroller and Auditor General, *Protecting and supporting the clinically extremely vulnerable during lockdown* HC 1131 (2021) paras 2.12‐2.13.

219 Office for National Statistics, ‘Coronavirus and redundancies in the UK labour market: September to November 2020’ 19 February 2021 at https://www.ons.gov.uk/employmentandlabourmarket/peopleinwork/employmentandemployeetypes/articles/labourmarketeconomicanalysisquarterly/december2020 (last visited 27 October 2021).

220 P. Inman, ‘Black, Asian and minority‐ethnic UK workers hit worst by Covid job cuts’ *The Guardian* 19 January 2021) at https://www.theguardian.com/business/2021/jan/19/black‐and‐minority‐ethnic‐uk‐workers‐hit‐worst‐by‐covid‐job‐cuts (last visited 27 October 2021). .

221 HC Deb vol 701 col 666 (Munira Wilson, Liberal Democrats) 19 October 2021; HC Deb vol 701 col 669 19 October 2021 (Neale Hanvey, Alba Party). MPs also expressed concern about wrongful prosecutions under Schedule 21 (HC Deb vol 701 col 666 19 October 2021 (Munira Wilson MP, Liberal Democrats)), the impact of cutting universal credit (HC Deb vol 701 col 655 19 October 2021 (Jonathan Ashworth MP, Labour)), and avoidable deaths during the pandemic (HC Deb vol 701 col 668 19 October 2021 (Richard Burgon MP, Labour), HC Deb vol 701 col 665 19 October 2021 (David Davis MP, Conservative)).

222 Joint Committee on Human Rights, n 189 above, 185‐198.

223
*ibid*, 178.

224 Coronavirus Act 2020, s 99(2)(b).

225 HL Deb vol 811 cols 984‐1048 25 March 2021.

226 K. Lines, 18 Months of COVID‐19 Legislation in England: A Rule of Law Analysis, (Bingham Centre for the Rule of Law, 2021); Lock, Grez Hidalgo and de Londras, n 176 above.

227 HC Deb vol 691 cols 1114 25 March 2021.

228 Coronavirus Act 2020, s 98.

229 See n 197 above.

230 The Coronavirus (Rights and Support) Bill (Liberty, 2021). For the purposes of disclosure the authors collaborated with Liberty on one element of the proposed Bill (a COVID‐specific select committee with express reviewing functions).

231 HC Deb vol 691 cols 1163‐1166 25 March 2021.

232 Described by de Londras in a different context as bottom‐up and top‐down panic: F. de Londras, *Detention in the War on Terror: Can Human Rights Fight Back?* (Cambridge: Cambridge University Press, 2011) ch 1.

233 For example, see Gross, n 10 above; Ackerman, n 10 above; Tushnet, n 10 above.

234 Dyzenhaus, n 10 above, 42, 205.

235 For example, in *Adiatu* v *Her Majesty's Treasury* [2020] EWHC 1554 (Admin) at [82], the High Court found regulations passed under the CVA to be ‘plainly justified’ on the basis of ‘applying the test of a wide margin of discretion’ afforded to the Government due to the subject matter concerning public finances.. For further discussion of judicial deference throughout the pandemic see Mackenzie‐Gray Scott, n 44 above.

236 Police Crime, Sentencing and Courts Act 2022, ss 198‐201.

237 Judicial Review and Courts Act 2022, s 42.

238 Coronavirus (Recovery and Reform) (Scotland) Bill at https://www.parliament.scot/bills‐and‐laws/bills/coronavirus‐recovery‐and‐reform‐scotland‐bill (last visited 11 April 2022).

239 Horsley, n 48 above.

240 The Joint Committee on Human Rights published its report *The Government's Response to COVID‐19* HC 265, HL 125 (2020) on the 21 September 2020, just days before the first SMR debate and vote. It was never mentioned in the debate. (HC Deb vol 681 cols 388‐415 30 September 2020). Similarly, the Women and Equalities Committee had published three reports, namely *Unequal impact? Coronavirus and BAME people* HC 284 (2020), *Unequal impact? Coronavirus, disability and access to services: full Report* HC 1050 (2020), *Unequal impact? Coronavirus and the gendered economic impact* HC 385 (2021) by SMR2. This work got only one mention, by the Chair of the Committee, at the SMR2 debate (HC Deb vol 691 col 1159 25 March 2021). These are just examples of the lack of impact in parliamentary debates of more than 70 parliamentary committee inquiries into Covid that had little, if any, impact on the six months reviews under the CVA.

241 Throughout the pandemic, the National Audit Office published some 30 reports between the 21 May 2020 and the 30 March 2020. We made references to some relevant reports in note 198 above. We also made references to reports produced by other public bodies in notes 199 and 200; and to NGO, academics and other organisations in notes 201, 207, 208, 210, 211, 216 and 217 above. These are all examples of the wide array of evidence available at the time of the various six months review processes for MPs to substantiate their scrutiny work.

242 See, for instance, references contained in note 105 above.

243 HC Deb vol 674 col 59 23 March 2020.

244
*R (Dolan)* v *Secretary of State for Health and Social Care* [2020] EWCA Civ 1605.

245
*Leigh and others* v *The Commissioner of the Police of the Metropolis and other* [2021] EWHC 661 (Admin).

246 Horsley, n 48 above, 111‐113.

247
*Dolan* n 244 above at [41]‐[42], [99].

248
*ibid* at [86]‐[90], [97], [110].

249
*Leigh* n 245 above.

250 HC Deb vol 691 col 1121 25 March 2020.

251
*Leigh* v *The Commissioner of Police of the Metropolis* [2022] EWHC 527 (Admin).

252 SPICe, ‘Consent processes in the Scottish Parliament’ (Scottish Parliament, 2021) at https://www.parliament.scot/‐/media/files/spice/factsheets/parliamentary‐business/consent‐processes‐in‐the‐scottish‐parliament‐14‐april‐2021.pdf (last visited 4 November 2020).

253 Royal Commission on the Reform of the House of Lords, *A House for the Future* Cm 4534 (2000) ch 4; Joint Committee on Conventions, *Conventions of the UK Parliament* HL 265‐I, HC 1212‐I (2006); D. Oliver, *Constitutional Guardians: The House of Lords* (London: The Constitution Society, 2016) 58‐60.

254 However, as the Scottish experience shows, extensive use of the MAP in the context of lockdown regulations has the ability to undermine the capacity of devolved legislatures to hold the government to account for lockdown policies. See Grez Hidalgo, de Londras and Lock, n 39 above.

255 Northern Ireland Assembly Hansard vol 127 No 5 7, 10, 12, 14, 15, 27, 29 24 March 2020; Senedd Record cols 276‐278, 306, 311‐312, 323, 331 24 March 2020; Scottish Parliament Official Report col 59‐60, 64, 70 24 March 2020.

256 Northern Ireland Assembly Hansard vol 127 No 5 25 (Martina Anderson MLA), 30 (Rachel Woods MLA), 35 (Gerry Carroll MLA) 24 March 2020; Scottish Parliament Official Report col 69 24 March 2020 (Anas Sarwar MSP).

257 For an account, see Blick and Hennessey, n 9 above. See Oliver, n 9 above.

258 See Oliver*, ibid*.

259
*R (Miller)* v *The Prime Minister* [2019] UKSC 41 at [48] (*Miller*).

260 For example, literature advocating effective legislative scrutiny of government bills is underpinned by the conviction that political actors ought to respect principles of liberal constitutionalism: D. Oliver, ‘Improving the Scrutiny of Bills: The Case for Standards and Checklists' (2006) PL 219; R. Hazell and D. Oliver, ‘The Constitutional Standards of the Constitution Committee: How a Code of Constitutional Standards Can Help Strengthen Parliamentary Scrutiny’ Constitution Unit, 2017 at https://constitution‐unit.com/2017/11/22/the‐constitutional‐standards‐of‐the‐constitution‐committee‐how‐a‐code‐of‐constitutional‐standards‐can‐help‐strengthen‐parliamentary‐scrutiny/ (last visited 14 June 2022); D. Feldman, ‘Parliamentary Scrutiny of Legislation and Human Rights’ (2002) PL 323; D. Feldman, ‘The Impact of Human Rights on the UK Legislative Process’ (2004) 25 *Statute Law Review* 91. Similarly, Elliot distinguishes between the legality and constitutionality of legislation: M. Elliott, ‘The Principle of Parliamentary Sovereignty in Legal, Constitutional, and Political Perspective’ in D. Oliver, J. Jowell and C. O'Cinneide (eds), *The Changing Constitution* (Oxford: OUP, 8th ed, 2015); M. Elliott, ‘Legislative Supremacy in a Multidimensional Constitution’ in D. Feldman and M. Elliott (eds), *Cambridge Companion to Public Law* (Cambridge: Cambridge University Press, 2016). cf Stephen Tierney and Martin Loughlin, who rather than embracing a morally committed theory of the UK constitution, describe these principles as constitutional facts which are expressions of changing power relationships between constitutional actors, and that operate as constraints on the political legitimacy of the otherwise theoretically supreme legislature. See M. Loughlin and S. Tierney, ‘The Shibboleth of Sovereignty’ (2018) 81 MLR 989.

261
*Miller* n 259 above at [41]‐[45]. By some, better conceptualised as ‘legislative supremacy’ (see Loughlin and Tierney, *ibid*).

262
*Miller ibid* at [46]‐[51].

263 A. Young, ‘The Relationship Between Parliament, The Executive and The Judiciary’ in J. Jowell and C. O'Cinneide (eds), *The Changing Constitution* (Oxford: OUP, 9th ed, 2019); R. Masterman, *The Separation of Powers in the Contemporary Constitution: Judicial Competence and Independence in the United Kingdom* (Cambridge: Cambridge University Press, 2010); *Duport Steels Ltd* v *Sirs* [1980] 1 All ER 529; *R* v *Secretary of State for the Home Department, ex p Fire Brigades Union* [1995] 2 All ER 244, 567.

264
*R (UNISON)* v *Lord Chancellor* [2017] UKSC 5; *X* v *Morgan‐Grampian Ltd* [1991] AC1 at [48]; *R (Cart)* v *Upper Tribunal* [2010] EWCA Civ 859 at [38].

265
*Entick* v *Carrington* (1795) 19 St Tr 1030; *Liversidge* v *Anderson* [1942] AC 206; *R* v *Secretary of State for the Home Department, ex parte Leech* [1999] QB 198; *British Oxygen* v *Minister for Technology* [1971] AC 610.

266 Judge, n 9 above.

267 Ewing, n 13 above, 1.

268 Lock, de Londras and Grez Hidalgo, n 39 above; Grez Hidalgo, de Londras and Lock, n 39 above.

269 R. Fox and J. Blackwell, *The Devil is in the Detail: Parliament and Delegated Legislation* (London: Hansard Society, 2014); R. Fox, J. Blackwell and B. Fowler, *Taking Back Control for Brexit and Beyond* (London: Hansard Society, 2017); A. Tucker, ‘Parliamentary Scrutiny of Delegated Legislation’ in A. Horne and G. Drewry (eds), *Parliament and the Law* (Oxford: Hart Publishing, 2nd ed, 2018).

270 See the various contributions and country reports in the two symposiums convened by J. Grogan: ‘COVID 19 and States of Emergency’ *Verfassungsblog* 2020 at https://verfassungsblog.de/category/debates/covid‐19‐and‐states‐of‐emergency‐debates/ and ‘Power and the COVID‐19 Pandemic’ *Verfassungsblog* 2021) at https://verfassungsblog.de/power‐and‐the‐covid‐19‐pandemic/ (both last visited 14 June 2022). See also C. Ayala Corao, ‘Challenges that the Covid‐19 Pandemic Poses to the Rule of Law, Democracy, and Human Rights’ Max Planck Institute for Comparative Public Law & International Law, Research Paper No 2020‐23; Chaplin, n 7 above; T. Ginsburg and M. Versteeg, ‘The bound executive: Emergency powers during the pandemic’ (2021) *International Journal of Constitutional Law* at https://doi.org/10.1093/icon/moab059 (last visited 9 November 2021).

271 For example, T. Ginsburg and A. Huq, *How to Save a Constitutional Democracy* (Chicago, IL: University of Chicago Press, 2018). In the UK context see A. Young, ‘Populism, and the UK Constitution’ (2018) 71 CLP 17.

272 A. Tomkins, *The Constitution after Scott – Government Unwrapped* (Oxford: Clarendon Press, 1998); K. Ewing and C. Gearty, *Freedom Under Thatcher* (Oxford: OUP, 1990).

